# Differentiating Associations of Glycemic Traits With Atherosclerotic and Thrombotic Outcomes: Mendelian Randomization Investigation

**DOI:** 10.2337/db21-0905

**Published:** 2022-10-01

**Authors:** Shuai Yuan, Amy M. Mason, Stephen Burgess, Susanna C. Larsson

**Affiliations:** 1 Unit of Cardiovascular and Nutritional Epidemiology, Institute of Environmental Medicine, Karolinska Institutet, Stockholm, Sweden; 2 British Heart Foundation Cardiovascular Epidemiology Unit, Department of Public Health and Primary Care, University of Cambridge, Cambridge, U.K.; 3 MRC Biostatistics Unit, University of Cambridge, Cambridge, U.K.; 4 Department of Public Health and Primary Care, University of Cambridge, Cambridge, U.K.; 5 Unit of Medical Epidemiology, Department of Surgical Sciences, Uppsala University, Uppsala, Sweden

## Abstract

We conducted a Mendelian randomization analysis to differentiate associations of four glycemic indicators with a broad range of atherosclerotic and thrombotic diseases. Independent genetic variants associated with fasting glucose (FG), 2 h glucose after an oral glucose challenge (2hGlu), fasting insulin (FI), and glycated hemoglobin (HbA_1c_) at the genome-wide significance threshold were used as instrumental variables. Summary-level data for 12 atherosclerotic and 4 thrombotic outcomes were obtained from large genetic consortia and the FinnGen and UK Biobank studies. Higher levels of genetically predicted glycemic traits were consistently associated with increased risk of coronary atherosclerosis–related diseases and symptoms. Genetically predicted glycemic traits except HbA_1c_ showed positive associations with peripheral artery disease risk. Genetically predicted FI levels were positively associated with risk of ischemic stroke and chronic kidney disease. Genetically predicted FG and 2hGlu were positively associated with risk of large artery stroke. Genetically predicted 2hGlu levels showed positive associations with risk of small vessel stroke. Higher levels of genetically predicted glycemic traits were not associated with increased risk of thrombotic outcomes. Most associations for genetically predicted levels of 2hGlu and FI remained after adjustment for other glycemic traits. Increase in glycemic status appears to increase risks of coronary and peripheral artery atherosclerosis but not thrombosis.

Atherosclerotic and thrombotic diseases, including coronary artery disease, ischemic stroke, peripheral artery disease, chronic kidney disease, and venous thromboembolism, are leading causes of global mortality and disease burden (1–3). Diabetes has been revealed to be pathological basis for atherosclerosis and thrombosis (4,5). High glycemic status, e.g., high blood glucose levels, has been found to partly explain the role of diabetes in the development and progression of atherosclerotic and thrombotic outcomes (4,5). Furthermore, glycemic traits have been found to be associated with these diseases in individuals without diabetes (6), which implies an important role of these commonly examined clinical indicators as potential predictors of subsequent atherosclerotic and thrombotic morbidity in both populations with diabetes and populations without diabetes. However, these associations were mainly founded on observational study designs, based on which one cannot infer causality due to possible confounding, misclassification, and reverse causality. A clear appraisal of causal associations of glycemic traits with atherosclerotic and thrombotic events can not only deepen understanding of pathology in these diseases but also determine predictive roles of glycemic traits for these outcomes.

Leveraging genetic variants as instrumental variables for an exposure, Mendelian randomization (MR) analysis can strengthen the causal inference in an exposure-outcome association (7). The analysis can minimize residual confounding because genetic variants are randomly assorted at conception and therefore not associated with environmental and self-adopted factors. MR analysis can also diminish, but not totally eliminate, reverse causality, since germline genotype cannot be modified by the onset or progression of the disease (8). In previous MR studies investigators have found a consistent influence of certain glycemic traits, such as glucose and glycated hemoglobin (HbA_1c_), on risk of coronary artery disease (9–13). Nevertheless, results of studies of the associations of glycemic traits with other atherosclerotic diseases, such as ischemic stroke (13–16) and chronic kidney disease (17–19), were inconsistent, and there is a scarcity of data on the MR associations for peripheral artery disease as well as other atherosclerotic and thrombotic outcomes (13).

Four glycemic traits, including fasting glucose (FG), 2 h glucose after an oral glucose challenge (2hGlu), fasting insulin (FI), and HbA_1c_, are commonly examined in the clinical setting. It has been hypothesized that different glycemic traits may be varyingly associated with atherosclerotic and thrombotic outcomes. A clear appraisal of causality of these associations is of great importance in detecting and managing cardiovascular risk in individuals with abnormal levels of glycemic traits. Here, we conducted a two-sample MR study with the aim of differentiating the associations of four glycemic traits with a broad range of atherosclerotic and thrombotic conditions. We also conducted multivariable MR analysis to explore the comparative roles of the four traits in relation to risk of atherosclerotic and thrombotic outcomes.

## Research Design And Methods

### Study Design

This MR study was based on publicly available summary-level data from large consortia and the UK Biobank and FinnGen studies. We estimated the associations of genetically predicted levels of four glycemic traits with 12 atherosclerotic and 4 thrombotic outcomes in single data sets and then combined estimates from different data sources. All studies included in cited genome-wide association studies had been approved by a relevant review board. All participants had provided inform consent. The present MR analyses were approved by the Swedish Ethical Review Authority, Uppsala, Sweden (2019-02793).

### Genetic Instrument Selection

Genetic variants (i.e., single nucleotide polymorphism [SNPs]) associated with FG, 2hGlu, FI, and HbA_1c_ at the genome-wide significance level (*P* < 5 × 10^−8^) were available in the Meta-Analysis of Glucose and Insulin-related Traits Consortium (MAGIC) with up to 196,991 individuals of European ancestry without diabetes (20). Linkage disequilibrium among these genetic variants was calculated with use of the 1000 Genomes European reference panel. SNPs with linkage disequilibrium (*r*^2^ > 0.001 or clump window [i.e., the distance between SNPs] <10 Mb) were removed, leaving 71 SNPs as instrumental variables for FG, 14 for 2hGlu, 38 for FI, and 75 for HbA_1c_ (Supplementary Table 1). These SNPs explain on average 3.6% phenotypic variance for FG, 0.8% for 2hGlu, 0.5% for FI, and 5.0% for HbA_1c_. Another set of SNPs was created with inclusion of all used SNPs for four glycemic traits. After exclusion of SNPs in linkage disequilibrium, 138 independent SNPs were used as instrumental variables in multivariable MR analysis. β-Coefficients and corresponding SEs for glycemic trait–SNP associations were obtained from genome-wide association analyses in European populations (20). The association tests were adjusted for BMI (except for HbA_1c_), study-specific covariates, and principal components (20). Adjustment for BMI in the genome-wide association analysis for FG, 2hGlu, and FI may introduce collider bias in MR analysis. We therefore obtained SNP–glycemic traits associations for used instrumental variables (IVs) from a previous genome-wide association study without adjustment for BMI (21).

### Outcome Data Sources

We obtained summary-level data for the associations of glycemic trait–associated SNPs with 12 atherosclerotic outcomes (coronary artery disease, angina, coronary atherosclerosis, coronary revascularization, ischemic stroke and its three subtypes, transient ischemic attack, aortic aneurysm, peripheral artery disease, and chronic kidney disease) and 4 thrombotic outcomes (subarachnoid hemorrhage, venous thromboembolism and its two subtypes, deep vein thrombosis, and pulmonary embolism) from large-scale genetic consortia (22–25), the FinnGen consortium (R5 data release) (26), and the UK Biobank study. There was no sample overlap between glycemic trait data and the FinnGen consortium or the UK Biobank study. Detailed information on outcome data sources (e.g., numbers of case and control subjects and covariates adjusted for in the genome-wide association analysis model) can be found in Supplementary Table 2. Descriptions of the FinnGen consortium and the UK Biobank study can be found in Supplementary Method, and outcome definitions for these two data sets are presented in Supplementary Table 3.

### Statistical Analysis

The inverse variance–weighted method with multiplicative random effects was used as the main statistical analysis method and supplemented by weighted median, MR-Egger regression, and contamination mixture analysis. The inverse variance–weighted analysis can provide the most precise estimates; however, it is sensitive to horizontal pleiotropy. Estimates of inverse variance–weighted analysis for one association from different sources were combined under a fixed-effects model. The weighted median method can generate consistent causal estimates when more than one-half of weight derives from valid genetic instruments (27). MR-Egger regression can provide estimates with adjustment for horizontal pleiotropy, even though the analysis usually consumes statistical power (28). The contamination mixture method can provide estimates in analysis using a large number of SNPs as instrumental variables with the presence of invalid SNPs (29). Considering correlations across glycemic traits and the aim of determining the comparative roles of glycemic traits in atherosclerotic and thrombotic outcomes, we performed multivariable MR to mutually adjust for four glycemic traits in one analysis. Similarly, estimates from different sources were combined with use of a fixed-effects model. In addition, we performed multivariable MR analysis for HbA_1c_ with adjustment for genetically predicted red blood cell distribution width using data from a study including 116,666 European individuals (30), given that HbA_1c_ appears to be associated with red blood cell–related traits, which may affect cardiovascular risk (31).

The F statistic was calculated to assess the strength of the instruments (Supplementary Table 4). The formula used for F statistic calculation was as follows:

$$\frac{N-k-\text{I}}{k}*\frac{R^{2}}{\text{I}-R^{2}}$$

where *N*, *k*, and *R*^2^ indicate sample size, number of instruments, and variance explained by instruments, respectively. Cochran *Q* value was used to assess the heterogeneity in estimates of SNPs for one association, and the *P* value for MR-Egger intercept was used as an indication of horizontal pleiotropy (*P* < 0.05). The false discovery rate method was used to correct for multiple testing (Supplementary Table 5). The association with a Benjamini-Hochberg–adjusted *P* value <0.05 was regarded as a significant association. The association with the original *P* value <0.05 and corrected *P* value >0.05 was deemed a suggestive association. All statistical tests were two sided and performed with the TwoSampleMR and MendelianRandomization packages (32,33) in R 4.0.2.

## Results

### FG

**Figure 1:**
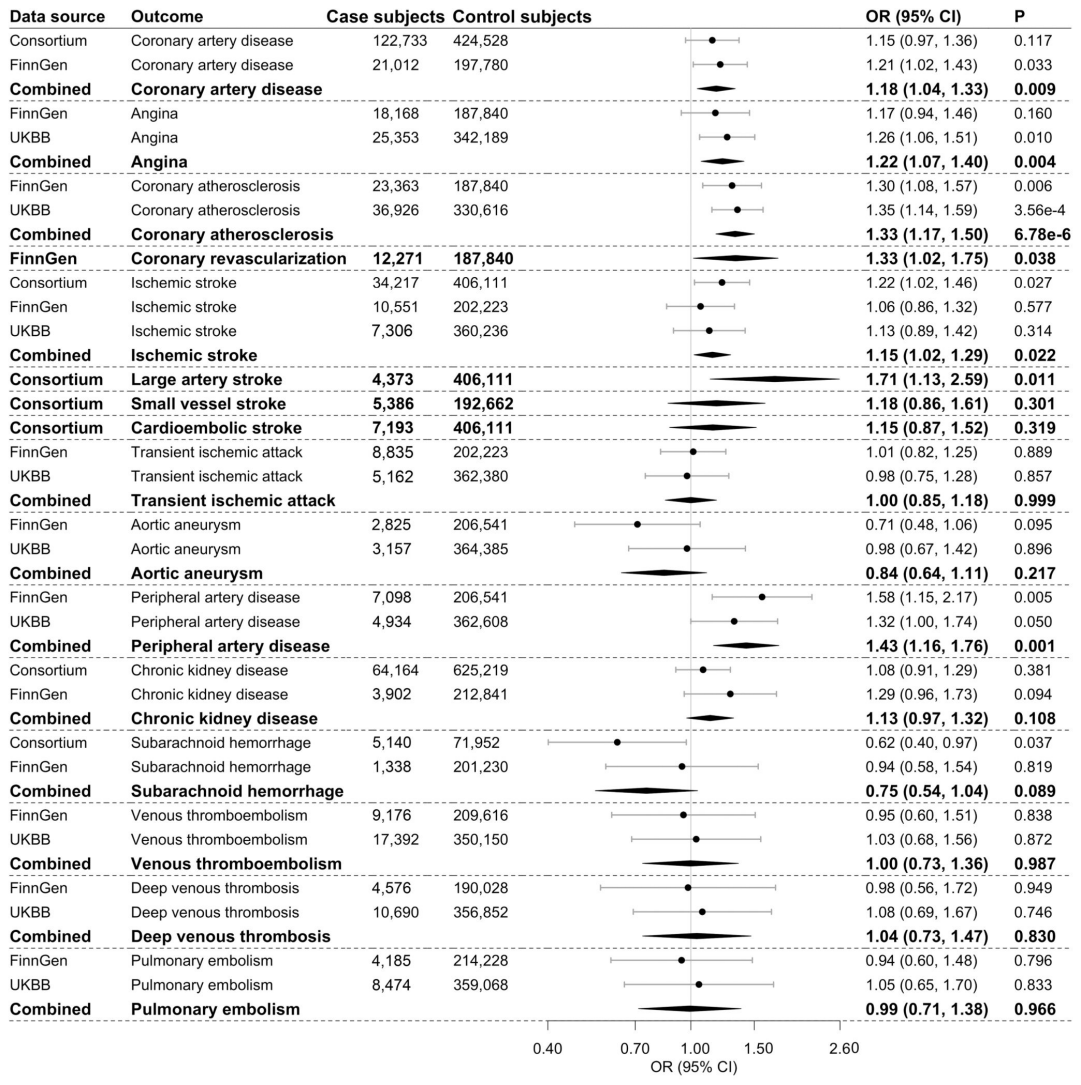
Associations of genetically predicted FG with atherosclerotic and thrombotic outcomes. The combined estimates are indicated with boldface type. The ORs were scaled to 1 mmol/L increase in genetically predicted FG. UKBB, UK Biobank.

Genetically predicted FG levels were associated with risk of coronary artery disease, angina, coronary artery atherosclerosis, coronary revascularization, ischemic stroke and its subtype large artery stroke, and peripheral artery disease (Fig. 1). The associations survived with the exception of coronary revascularization and ischemic stroke after false discovery rate correction (Supplementary Table 5). For each 1 mmol/L increase in genetically predicted FG, the odds ratio (OR) was 1.18 (95% CI 1.04, 1.33) for coronary artery disease, 1.22 (1.07, 1.40) for angina, 1.33 (1.17, 1.50) for coronary atherosclerosis, 1.71 (1.13, 2.59) for large artery stroke, and 1.43 (1.16, 1.76) for peripheral artery disease. There were suggestive associations for coronary revascularization (OR 1.33; 95% CI 1.02, 1.75) and ischemic stroke (1.15; 1.02, 1.29). Genetically predicted FG levels were not associated with other outcomes (Fig. 1).

### 2hGlu

**Figure 2:**
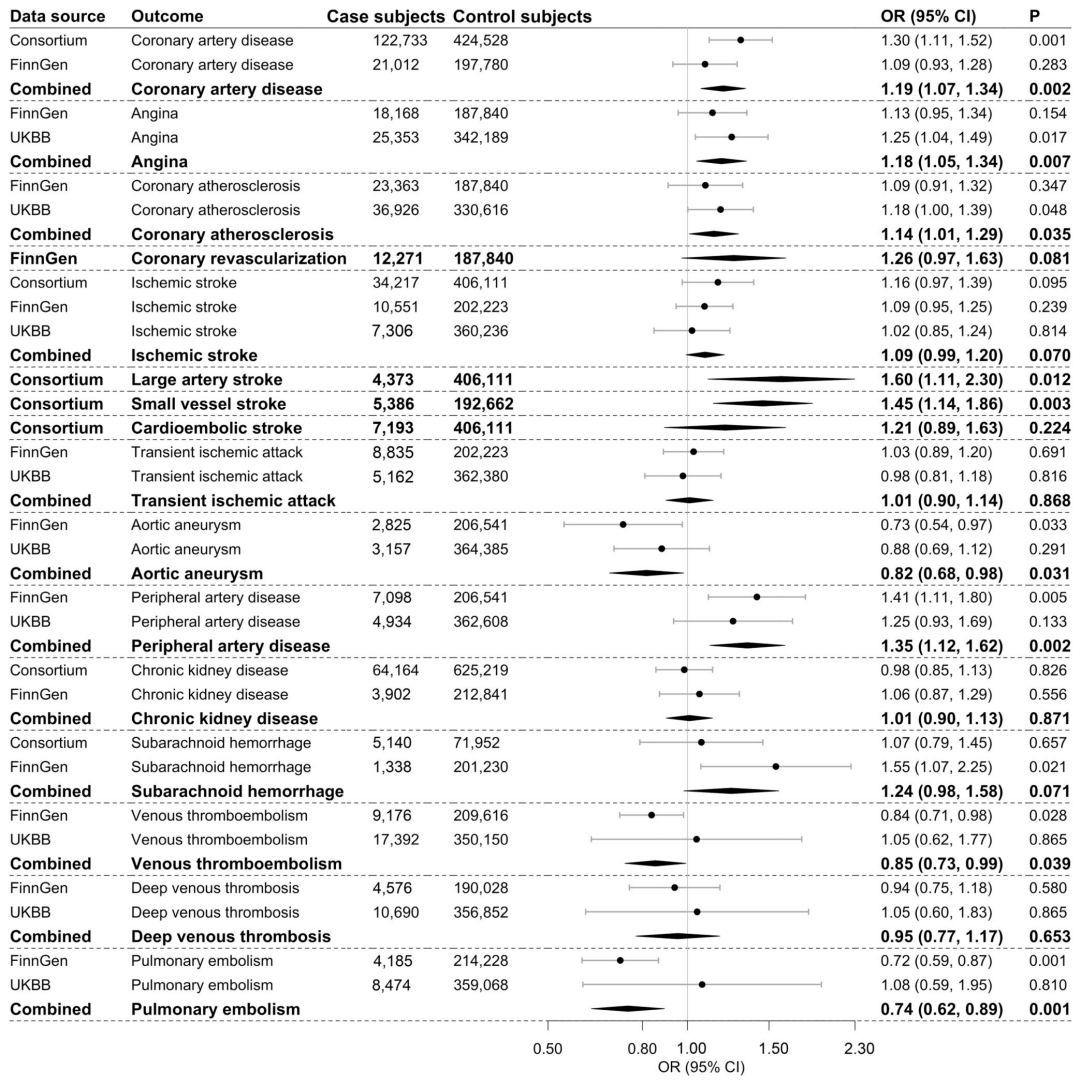
Associations of genetically predicted 2hGlu with atherosclerotic and thrombotic outcomes. The combined estimates are indicated with boldface type. The ORs were scaled to 1 unit in log-transformed millimoles per liter increase in genetically predicted levels of 2hGlu. UKBB, UK Biobank.

Genetically predicted 2hGlu levels were associated with nine atherosclerotic and thrombotic outcomes (Fig. 2). After multiple testing correction, higher genetically predicted 2hGlu levels were associated with increased risk of coronary artery disease (OR per 1 unit in log-transformed millimoles per liter increase, 1.19; 95% CI 1.07, 1.33), angina (1.18; 1.05, 1.34), large artery stroke (1.60; 1.11, 2.30), small vessel stroke (1.45; 1.14, 1.86), and peripheral artery disease (1.35; 1.12, 1.62) and associated with decreased risk of pulmonary embolism (0.74; 0.62, 0.89). There were suggestive associations for coronary atherosclerosis (1.14; 1.01, 1.29), aortic aneurysm (0.82; 0.68, 0.98), and venous thromboembolism (0.85; 0.73, 0.99).

### FI

**Figure 3:**
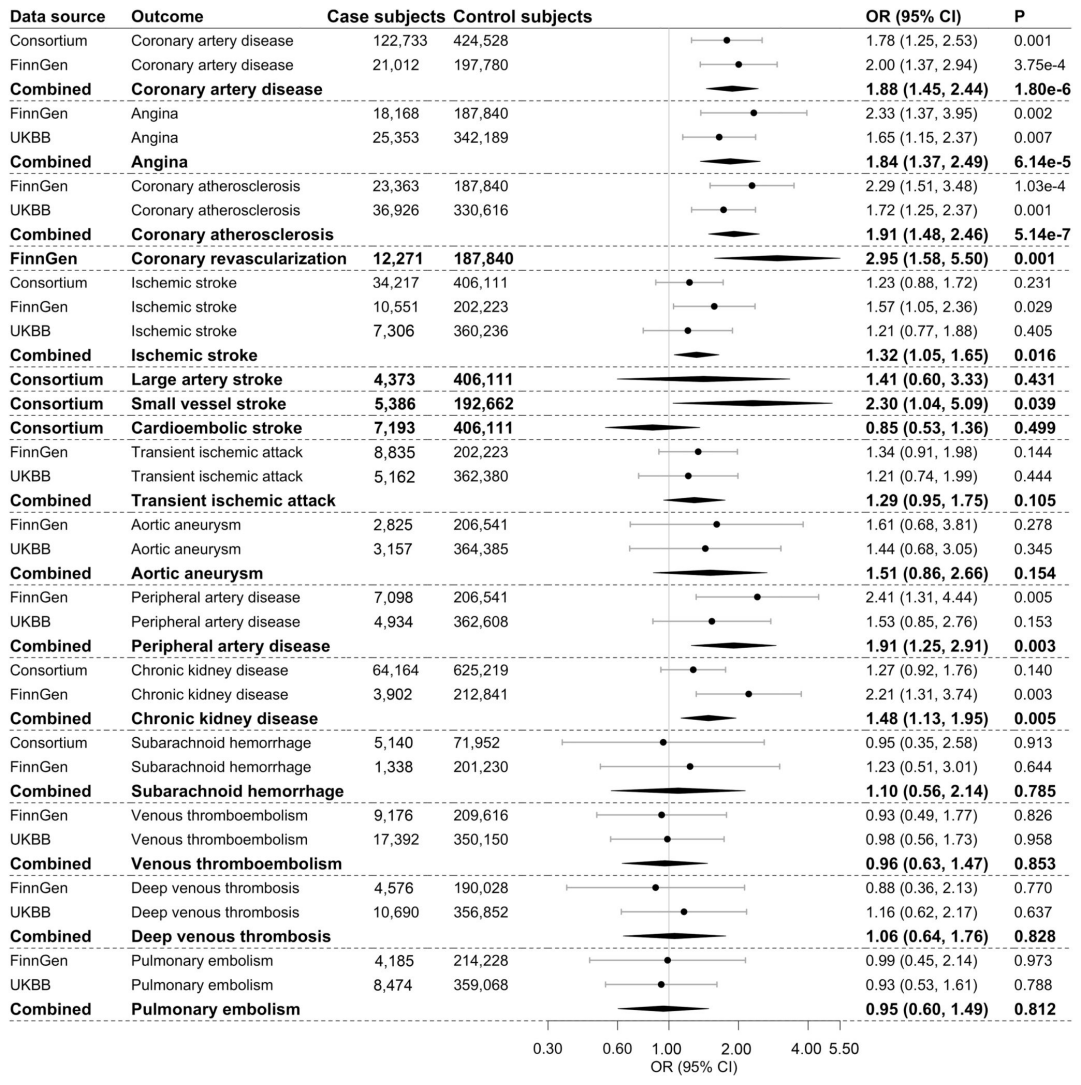
Associations of genetically predicted FI with atherosclerotic and thrombotic outcomes. The combined estimates are indicated with boldface type. The ORs were scaled to 1 unit in log-transformed picomoles per liter increase in genetically predicted FI levels. UKBB, UK Biobank.

Genetically predicted FI levels were associated with coronary artery disease, angina, coronary atherosclerosis, coronary revascularization, ischemic stroke, small vessel stroke, peripheral artery disease, and chronic kidney disease (Fig. 3). The association for small vessel stroke became suggestive after multiple testing correction (Supplementary Table 5). Per 1 unit in log-transformed picomoles per liter increase in genetically predicted FI levels, the OR was 1.88 (95% CI 1.45, 2.44) for coronary artery disease, 1.84 (1.37, 2.49) for angina, 1.91 (1.48, 2.46) for coronary atherosclerosis, 2.95 (1.58, 5.50) for coronary revascularization, 2.30 (1.04, 5.09) for small vessel stroke, 1.91 (1.25, 2.91) for peripheral artery disease, and 1.48 (1.13, 1.95) for chronic kidney disease. There were limited data in support of any associations with other atherosclerotic or thrombotic outcomes (Fig. 3).

### HbA_1c_

**Figure 4:**
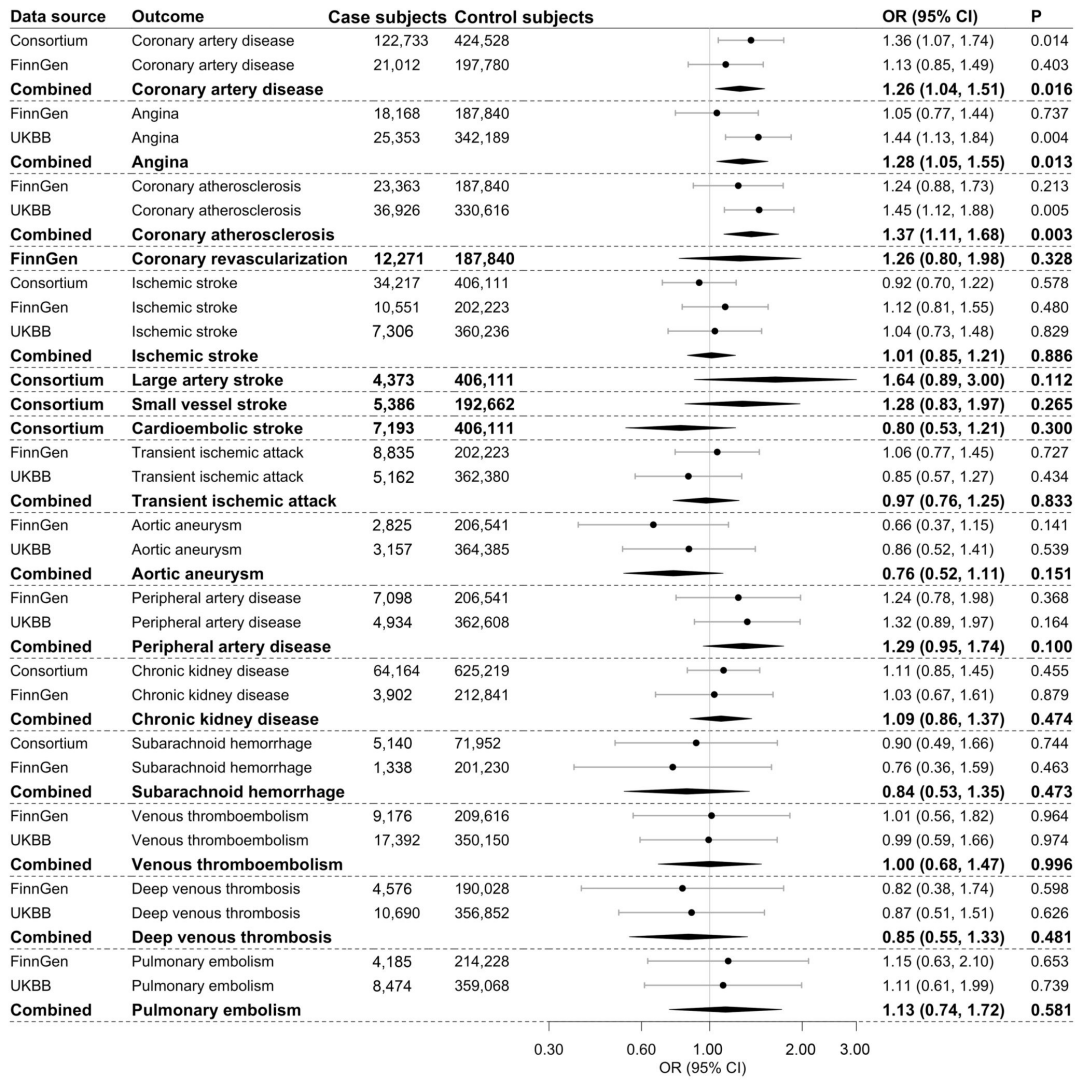
Associations of genetically predicted HbA_1c_ with atherosclerotic and thrombotic outcomes. The combined estimates are indicated with boldface type. The ORs were scaled to 1 percentage increase in genetically predicted HbA_1c_ levels. UKBB, UK Biobank.

Genetically predicted HbA_1c_ levels were significantly associated with coronary atherosclerosis and suggestively associated with coronary artery disease and angina (Fig. 4). For 1 percentage increase in genetically predicted HbA_1c_ levels, the OR was 1.26 (95% CI 1.04, 1.51) for coronary artery disease, 1.28 (1.05, 1.55) for angina, and 1.37 (1.11, 1.68) for coronary atherosclerosis. Genetically predicted HbA_1c_ levels were not associated with other outcomes (Fig. 4). The associations slightly changed in multivariable MR with adjustment for genetically predicted red blood cell distribution width (Supplementary Table 6).

### Sensitivity Analyses

Associations of genetically predicted glycemic traits with the studied outcomes were overall consistent in the weighted median and contamination mixture analyses, but the CIs were broad in MR-Egger regression analyses (Supplementary Table 7). We observed moderate-to-high heterogeneity in most analyses; however, no pleiotropy was observed with the exception of a few MR-Egger analyses of FG and 2hGlu (Supplementary Table 7). Compared with results from the main analysis, the associations for genetically predicted FG, 2hGlu, and FI were overall consistent albeit with wider CIs (caused by a smaller sample size for genome-wide association analysis on glycemic traits and missing SNPs in the analysis) in the sensitivity analysis using SNP–glycemic trait estimates without adjustment for BMI (Supplementary Table 8).

### Multivariable MR Analyses

Most associations for genetically predicted levels of FG and HbA_1c_ attenuated and became statistically nonsignificant in multivariable MR analysis with adjustment for other glycemic traits after multiple testing correction (Supplementary Table 9). Genetically predicted levels of 2hGlu showed associations with coronary artery disease, angina, ischemic stroke, large artery stroke, small vessel stroke, and peripheral artery disease in the multivariable MR analysis (Supplementary Table 9). Genetically predicted levels of FI were associated with risk of coronary artery disease, angina, coronary atherosclerosis, coronary revascularization, transient ischemic attack, and chronic kidney disease in the multivariable MR analysis (Supplementary Table 9). A summary of findings from multivariable MR analysis can be found in Supplementary Fig. 1.

## Discussion

**Figure 5:**
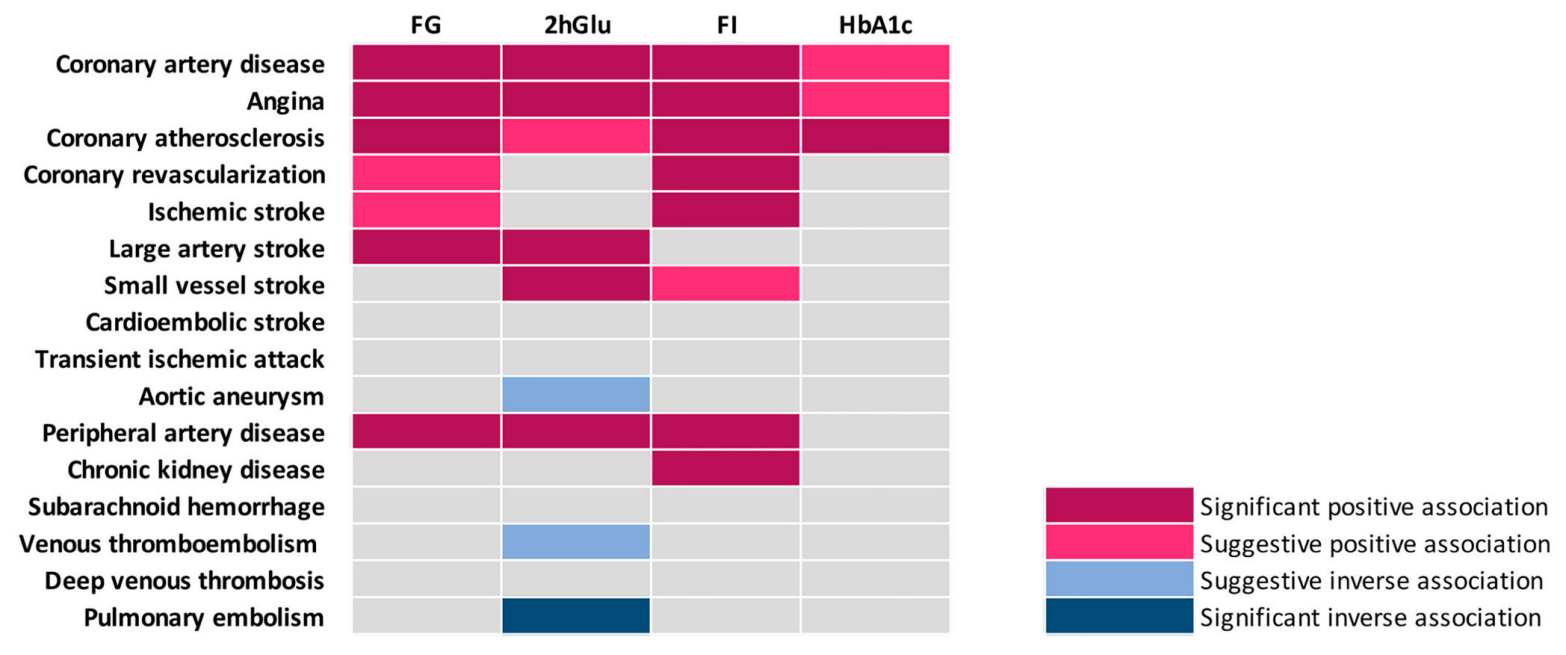
Summary of associations of genetically predicted glycemic traits with 12 atherosclerotic and 4 thrombotic outcomes.

This MR study systematically differentiated associations of four genetically predicted glycemic traits with 12 atherosclerotic and 4 thrombotic outcomes (Fig. 5). Our findings showed that higher levels of genetically predicted glycemic traits were consistently associated with increased risk of coronary atherosclerosis and its related diseases and symptoms. Genetically predicted glycemic traits except HbA_1c_ showed positive associations with peripheral artery disease risk. Genetically predicted FI levels were associated with risk of ischemic stroke and chronic kidney disease. Genetically predicted FG and 2hGlu were positively associated with risk of large artery stroke. Additionally, genetically predicted 2hGlu levels showed positive associations with risk of small vessel stroke. Higher levels of genetically predicted glycemic traits were not associated with increased risk of thrombotic outcomes. Instead, genetically predicted 2hGlu levels appeared to be inversely associated with pulmonary embolism. Most associations for genetically predicted levels of 2hGlu and FI remained after adjustment for other glycemic traits.

Associations of FG, FI, and HbA_1c_ with coronary artery disease have been found in population-based observational studies (34,35) and replicated in MR studies (9–13). Our updated MR analysis confirmed these associations with a larger number of instruments and greatly augmented sample sizes. We additionally found associations of these glycemic traits with other coronary artery atherosclerosis–related outcomes and symptoms, which further strengthened the casual role of high glycemic status in triggering coronary artery atherosclerosis. Except for FG, FI, and HbA_1c_, our study revealed novel causal associations of 2 h postchallenge glucose (i.e., 2hGlu) with a wide range of coronary artery atherosclerotic events.

Studies of glycemic traits in relation to risk of peripheral artery disease are scarce. In a cohort study of 11,634 participants followed up for 20.7 years, high FG and HbA_1c_ levels were associated with increased risk of peripheral artery disease (36). However, in a recent MR study based on 7,071 case subjects, no association was found between glucose levels proxied by seven SNPs and peripheral artery disease risk in participants without diabetes (19), but the nonsignificant positive association might be caused by inadequate power. In another MR study, a positive association was found between genetically predicted HbA1c levels and peripheral vascular disease in the UK Biobank study, but the association did not persist in individuals without diabetes (13). In the current MR investigation, higher genetically predicted levels of FG, 2hGlu, and FI, but not HbA1c, were consistently associated with an increased risk of peripheral artery disease. Along with findings regarding coronary artery end points, these results imply the universally detrimental role of high glycemic status in both coronary and peripheral artery atherosclerosis and the importance of glycemic management for preventing and delaying progression of related diseases.

FG and HbA_1c_ levels have been associated with elevated risk of ischemic stroke in observational studies (6,37). However, MR results of the association between FG and ischemic stroke are conflicting. In our previous MR study, with 36 SNPs for FG and 18 SNPs for FI, we did not detect any association with ischemic stroke on the basis of METASTROKE consortium data (16). Nevertheless, higher plasma glucose levels proxied by 10 SNPs were associated with increased risk of ischemic stroke in a subsequent MR meta-analysis study of Copenhagen studies and the METASTROKE consortium (15). In a recent MR study based on METASTROKE and many more SNPs for FG, FI, and HbA_1c_, clear associations were found of genetically predicted FI and HbA_1c_, but not FG, with ischemic stroke (14). The association for HbA_1c_ was not observed in a study with data from the UK Biobank study (13). Our MR of three data sources revealed positive associations of genetically predicted FG and FI levels with ischemic stroke, in particular, large-vessel stroke as well as small vessel stroke. In addition, we also observed associations of 2hGlu with large-vessel stroke and small vessel stroke. While the reasons for the discrepancy across the above-mentioned studies are unclear, it is likely caused by insufficient power and different genetic instrument selection.

Our null findings for genetically predicted FG and HbA_1c_ in relation to chronic kidney disease agree with the results of previous MR studies (18). Although we observed a positive association between genetically predicted FI and chronic kidney disease, this association seemed to be sex specific (17). Given lack of sex-specific data on chronic kidney disease in the used data sets, we could not confirm this sex-specific association.

We had several novel findings, mainly for 2hGlu, which was inversely associated with aortic aneurysm. Even though these associations have not previously been examined in MR analysis, these observed associations partly support observational findings on FG in relation to aortic aneurysm (38). In addition, 2hGlu levels are correlated with other glycemic traits. However, studies have suggested that 2 h postchallenge glucose might be a superior glycemic indicator for assessing risk of incident coronary artery disease in healthy populations (39). This hypothesis appears partly supported by findings from our multivariable MR analysis where the associations for 2hGlu but not for FG and HbA_1c_ remained after adjustment for other glycemic traits. The possible reason is that compared with FG and HbA_1c_, 2hGlu levels might be a better indicator for detecting intima-media thickness and capturing additional information on the effects of glycemic metabolism impairment on cardiovascular disease risk, especially in individuals with prediabetes or at the early diabetes stage (40). Likewise, our multivariable MR analysis showed robustness of associations for genetically predicted levels of FI, which might be used as an important glycemic feature for detecting risk of coronary artery atherosclerosis–related outcomes, stroke, and chronic kidney disease.

Even though type 2 diabetes has been associated with increased risk of venous thromboembolism (41), observational studies have provided limited evidence in support of any association of glycemic traits (42,43) with venous thromboembolism. In this MR study we did not find detrimental effects of glycemic traits on venous thromboembolism or its two subtypes, which suggests that high glycemic status does not play an important role in thrombosis.

There are several important cellular and molecular pathophysiologic pathways involved in the association between the impaired glycemic feature and increase risk of atherosclerotic cardiovascular disease, such as inflammation, increased oxidative stress, endothelial dysfunction, hypercoagulability, vascular calcification, and epigenetics (44,45). These detailed mechanisms have been thoroughly discussed in previous studies (44,45). In addition, high glycemic status may induce angiotensin II production and thus increase blood pressures as well as risk of atherosclerotic cardiovascular disease (46). The positive association between HbA_1c_ and blood pressure was observed in a recent MR study (47). For aortic aneurysm, high levels of 2hGlu appear to be protective. Although the underlying mechanisms supporting this inverse association have not been fully investigated, there are some hypotheses concerning increased synthesis and reduced degradation of extracellular matrix, increased collagen synthesis, greater abdominal aorta thickness, and decreased aortic wall stress and protease expression and activity in individuals with high glycemic status compared with those with normal levels of glycemic traits (48). A recent review revealed additional possible mechanisms including decreased neoangiogenesis, increased clot density, decreased porosity, and enhanced TGF-β signaling pathway in individuals with higher glycemic status (49). High glycemic status can also influence renewal of mural thrombosis via lowering fibrinolysis, which may lower risk of aortic aneurysm and venous thromboembolism (48). Other possible mechanisms explaining the inverse association between glucose levels and thrombosis have scarcely been revealed (50).

There are several strengths of the current study. The major one is the MR design, which can strengthen causal inference by diminishing residual confounding and reverse causality. Even though reverse causality may still bias MR associations (8), this issue should be minimal in current data, since there were few glycemic trait–associated SNPs directly and strongly associated with outcomes. In addition, underlying mechanisms have been well studied for certain associations. We examined the associations of glycemic traits with outcomes in different independent data sets. The consistent findings between different outcome data increased confidence in causal inference. In addition, combining different sources increased power to detect weak associations, even though we might still overlook associations for outcomes with a small number of cases. The study included a wide range of atherosclerotic outcomes. Although high glycemic status has been associated with atherosclerosis, our study added evidence to differentiate the associations of specific glycemic traits with different atherosclerotic end points, in particular, rarely studied outcomes. Population structure bias is expected to be small, as genetic data were obtained from individuals of European descent (except in the analysis for coronary artery disease in a consortium including a small proportion of individuals of non-European descent) and corresponding genome-wide association analyses were adjusted for genetic principal components.

Limitations need attention for interpretation of our findings. An important limitation is pleiotropy. SNPs associated with glycemic traits might be correlated with other metabolic factors, such as BMI and type 2 diabetes. The genome-wide association analyses on glycemic traits were adjusted for BMI (20). Thus, the effect of the used instrument should be independent of this factor and corresponding pleiotropy from obesity is likely to be minimal. In addition, if observed associations between genetic glycemic traits and atherosclerotic outcomes were driven by a pleiotropic effect from obesity, the inverse or neutral associations for thrombotic outcomes could not be justified, since obesity is a strong risk factor for thrombosis (51). Regarding diabetes, the associations of glycemic traits with studied outcomes should partly support and explain the associations between diabetes and these diseases. Moreover, we selected instruments from genome-wide association studies that included participants free of diabetes, which should reduce the pleiotropic effect from diabetes. The inclusion of individuals with diabetes in the outcome data sets might have had some influence on our results because it is unknown whether SNPs selected from individuals without diabetes can properly reflect the levels of glycemic traits in patients with diabetes. As for HbA_1c_, certain SNPs were associated with erythrocyte-related traits that may affect risk of cardiovascular disease (31). Even though the associations for HbA_1c_ changed slightly in the multivariable MR analysis with adjustment for one representative erythrocyte-related trait (i.e., red blood cell distribution width), we could not completely rule out the possibility that the observed associations for HbA_1c_ were not affected by other erythrocyte-related traits. However, given that HbA_1c_ and erythrocyte-related features are biologically correlated, the effects of erythrocyte-related features should be interpreted not as pleiotropy but as a part of the total effects of HbA_1c_. There might be some sample overlap in the analyses based on consortia data, which might bias causal estimates toward observational associations. However, any sample overlap unlikely had a major influence on our results for two reasons. First, we observed generally consistent results from analyses based on consortia and two other data sets without sample overlap with glycemic trait data. Second, F statistic >10 for all analyses with the exception of the analysis of FI based on consortium data (24) indicated a good strength of our genetic instruments and limited sample overlap bias (52). For certain outcomes, the definition of the outcome differed somewhat in the FinnGen and UK Biobank studies, and this might have caused heterogeneity in results. However, observed associations were generally consistent between the two sources, suggesting that any heterogeneity caused by differences in phenotypic definitions should be small. Phenotypic variances explained by used SNPs for 2hGlu and FI were small, even though we obtained data from the hitherto largest genome-wide association study on glycemic traits (20). The small variances for 2hGlu and FI might inflate the rate of false negatives, although we combined different data sources to increase the power. In addition, the nonlinearity of these associations could not be examined in this study due to summary-level data. The nonlinear associations should be tested in future studies, even though these associations are more likely to be of a linear fashion (12). The multivariable MR analysis relies on strong assumptions that are almost impossible to empirically test (53). Thus, the validity of multivariable MR analysis needs to be further assessed.

In conclusion, this MR study differentiates the roles of four glycemic traits in the development of a broad range of atherosclerotic and thrombotic outcomes. Our findings suggest that increased glycemic status is a risk factor for both coronary and peripheral artery atherosclerosis but not for thrombosis. Among glycemic traits, 2hGlu and FI appear to be good predictors of increased risk of coronary atherosclerosis and ischemic stroke.

## Supplementary Material

Supplementary File 1

## Data Availability

Analyses of UK Biobank data were performed under application 29202. The data sets analyzed except for UK Biobank in this study are publicly available summary statistics. Data used can be obtained upon a reasonable request to the corresponding author.
