# Supplementary material for: Differentiating Associations of Glycemic Traits With Atherosclerotic and Thrombotic Outcomes: Mendelian Randomization Investigation
Source: Diabetes. Author manuscript; Available in PMC 2022 Nov 27. (PMC7613853; doi:10.2337/db21-0905)
Supplement: Supplementary File 1 [file EMS157098-supplement-Supplementary_File_1.pdf]

Differentiating associations of glycemic traits with atherosclerotic and thrombotic outcomes: Mendelian randomization investigation

Shuai Yuan, Amy M. Mason, Stephen Burgess, Susanna C. Larsson

Major Resources Table

Supplementary method

Supplementary Table 1. Genetic instruments for glycemic traits
Supplementary Table 2. Outcome data information
Supplementary Table 3. Diagnostic information in FinnGen and UK Biobank
Supplementary Table 4. Strength of genetic instruments
Supplementary Table 5. Results of false discovery rate correction
Supplementary Table 6. Associations for HbA1c after adjusting for genetically predicted red blood cell distribution width
Supplementary Table 7. Associations of genetically predicted glycemic traits with atherosclerotic and thrombotic outcomes in sensitivity analysis
Supplementary Table 8. Comparison of associations for genetically predicted FG, 2hGlu, and FI in the main analysis and the sensitivity analysis using SNP-glycemic trait estimates without adjustment for body mass index
Supplementary Table 9. Associations of genetically predicted glycemic traits with atherosclerotic and thrombotic outcomes in multivariable Mendelian randomization analysis with mutual adjustment

Supplementary Figure 1. Summary of associations of genetically predicted glycemic traits with 12 atherosclerotic and 4 thrombotic outcomes in multivariable Mendelian randomization analysis.

Major Resources Table

Data & Code Availability

Description	Source / Repository	Persistent ID / URL
Codes	NA	It can be obtained upon a reasonable request to the corresponding author.
Data	Publicly available<br>UKB data can be applied through https://www.ukbiobank.ac.uk/	It can be obtained upon a reasonable request to the corresponding author.

Supplementary method

The UK Biobank study

The UK Biobank is a cohort study of approximately 500 000 adults aged 37 to 73 years of age in 2006-2010. In the present study, we excluded participants with non-European ethnicities (to reduce population stratification bias), those with relatedness of third degree or higher, excess heterozygosity, and low genotype call rate, leaving a sample of 367 561 individuals of European ancestry. The cardiovascular endpoints were defined by codes from the 9th and 10th versions of International Classification of Disease, procedure codes for surgery and self-reported information verified by interview with a nurse. Cases were ascertained until July 27, 2021. The numbers of cases ranged from 601 for thoracic aortic aneurysm to 35 979 for coronary artery disease. We estimated the genetic associations with cardiovascular disease using logistic regression with adjustment for age, sex, and 10 genetic principal components. The UK Biobank was approved by the North West Multicenter Research Ethics Committee. All participants provided written informed consent.

The FinnGen consortium

The FinnGen consortium is an ongoing study collecting information on health outcomes and genetic data from Finnish biobanks and digital health record data from Finnish health registries. In this study, we used the R5 data release of the FinnGen consortium, including up to 218,792 individuals. In this dataset, individuals with ambiguous gender, high genotype missingness (>5%), excess heterozygosity (±4 standard deviation) and non-Finnish ancestry had been excluded. Genetic variants with high missingness (>2%), low Hardy-Weinberg equilibrium p-value ($p<5\times10$-6) and minor allele count, minor allele counts <3 were excluded. Association tests had been adjusted for age, sex, 10 genetic principal components and genotyping batch. Cardiovascular cases were defined by codes from the 8th, 9th and 10th versions of International Classification of Disease and surgery and medicine purchase codes from nationwide registries.

Supplementary Table 1. Genetic instruments for glycemic traits

Exposure	rsID	Chr	Pos_hg19	EA	NEA	EAF	Beta	SE	P value
2hGlu	rs12692738	2	165558252	T	C	0.74	0.0486	0.0090	2.72E-08
2hGlu	rs1260326	2	27730940	T	C	0.41	0.0486	0.0078	5.93E-12
2hGlu	rs11708067	3	123065778	A	G	0.82	0.0872	0.0093	1.98E-22
2hGlu	rs144346724	3	185520085	G	GTGTTCTTGACTAACCAAGACA	0.75	-0.0608	0.0087	1.02E-11
2hGlu	rs878521	7	44255643	A	G	0.25	0.0990	0.0094	1.25E-28
2hGlu	rs2126259	8	9185146	T	C	0.09	-0.0716	0.0121	2.98E-10
2hGlu	rs550057	9	136146597	T	C	0.29	0.0526	0.0085	3.62E-11
2hGlu	rs7903146	10	114758349	T	C	0.31	0.0854	0.0087	2.79E-26
2hGlu	rs4148646	11	17415190	C	G	0.34	0.0397	0.0078	4.39E-08
2hGlu	rs2649999	12	121380544	T	C	0.36	0.0498	0.0082	2.01E-10
2hGlu	rs112824462	14	38842759	A	G	0.28	-0.0584	0.0102	6.73E-09
2hGlu	rs17271305	15	62332980	A	G	0.59	-0.0587	0.0077	2.88E-14
2hGlu	rs117643180	17	7185779	A	C	0.03	0.2340	0.0327	7.31E-14
2hGlu	rs1800437	19	46181392	C	G	0.21	0.1004	0.0099	4.79E-26
FG	rs6662924	1	100894419	A	C	0.20	0.0143	0.0023	3.34E-10
FG	rs78132593	1	150868102	A	C	0.20	-0.0147	0.0022	2.60E-10
FG	rs2075423	1	214154719	T	G	0.38	-0.0161	0.0017	3.18E-21
FG	rs348330	1	229672955	A	G	0.63	-0.0122	0.0020	3.04E-10
FG	rs7584277	2	169643225	A	G	0.08	0.0266	0.0036	1.61E-14
FG	rs180935712	2	169681425	A	G	0.01	0.0570	0.0082	2.12E-11
FG	rs140809953	2	169681654	A	G	0.02	0.0369	0.0073	2.11E-08
FG	rs13431652	2	169753415	T	C	0.68	0.0693	0.0018	1.00E-200
FG	rs13389076	2	169789512	A	G	0.03	0.0609	0.0049	1.72E-36
FG	rs1057394	2	27324036	A	G	0.63	-0.0124	0.0018	1.91E-12
FG	rs1260326	2	27730940	T	C	0.41	-0.0282	0.0017	4.48E-65
FG	rs77981966	2	43777964	T	C	0.06	-0.0246	0.0035	1.58E-14
FG	rs189548	2	54941112	A	G	0.72	-0.0123	0.0020	2.81E-09
FG	rs11708067	3	123065778	A	G	0.82	0.0281	0.0020	1.63E-43
FG	rs16851397	3	141134818	A	G	0.96	0.0327	0.0042	1.26E-12
FG	rs17437560	3	152180329	T	C	0.11	-0.0175	0.0032	3.33E-08
FG	rs1604038	3	170709193	T	C	0.29	-0.0198	0.0018	4.47E-28
FG	rs201104047	3	170736864	D	I	0.14	-0.0240	0.0029	1.26E-17
FG	rs6808574	3	187740523	T	C	0.39	-0.0127	0.0017	7.21E-14
FG	rs4862423	4	185726548	T	C	0.40	0.0123	0.0019	4.45E-10
FG	rs157512	5	55809127	T	C	0.73	0.0134	0.0021	5.43E-10
FG	rs7708285	5	76425867	A	G	0.69	-0.0133	0.0019	1.25E-09
FG	rs1820176	5	95696585	T	C	0.70	0.0247	0.0020	1.91E-34
FG	rs12055786	6	153431125	T	C	0.38	0.0120	0.0017	1.17E-11
FG	rs9348441	6	20680678	A	T	0.27	0.0176	0.0018	4.40E-20
FG	rs10305457	6	39034095	T	C	0.06	0.0235	0.0032	1.21E-14
FG	rs3778321	6	7250270	A	G	0.18	-0.0186	0.0021	3.16E-17
FG	rs17168486	7	14898282	T	C	0.18	0.0280	0.0021	4.17E-36
FG	rs10487796	7	15063430	A	T	0.48	-0.0261	0.0016	4.62E-52
FG	rs2595701	7	44148553	A	G	0.32	0.0189	0.0021	4.48E-19
FG	rs878521	7	44255643	A	G	0.25	0.0549	0.0020	2.65E-174

FG	rs58925536	7	75654574	T	C	0.03	0.0306	0.0053	5.82E-09
FG	rs194518	7	89853149	A	G	0.52	0.0102	0.0018	8.76E-09
FG	rs9650069	8	118204020	T	C	0.28	-0.0286	0.0018	8.31E-58
FG	rs12541643	8	81076874	T	C	0.48	0.0118	0.0019	4.51E-09
FG	rs7012637	8	9173209	A	G	0.47	-0.0180	0.0017	9.75E-25
FG	rs896854	8	95960511	T	C	0.46	0.0099	0.0016	5.61E-09
FG	rs16913693	9	111680359	T	G	0.97	0.0394	0.0049	2.82E-16
FG	rs507666	9	136149399	A	G	0.19	0.0164	0.0021	6.99E-17
FG	rs3829109	9	139256766	A	G	0.28	-0.0163	0.0020	1.09E-15
FG	rs10811660	9	22134068	A	G	0.17	-0.0223	0.0022	7.94E-25
FG	rs10974438	9	4291928	A	C	0.62	-0.0198	0.0017	9.85E-31
FG	rs12784552	10	113036354	A	G	0.92	0.0329	0.0030	2.86E-31
FG	rs7903146	10	114758349	T	C	0.31	0.0259	0.0019	2.00E-35
FG	rs2839671	10	26505822	A	G	0.16	-0.0160	0.0022	8.38E-14
FG	rs7095788	10	95384152	T	C	0.36	-0.0106	0.0018	1.98E-09
FG	rs3842753	11	2181060	T	G	0.28	0.0134	0.0022	2.84E-09
FG	rs10838524	11	45870177	A	G	0.48	0.0238	0.0016	1.56E-40
FG	rs10838693	11	47350553	C	G	0.31	0.0177	0.0018	3.44E-23
FG	rs174583	11	61609750	T	C	0.38	-0.0168	0.0017	3.37E-22
FG	rs11603349	11	72460694	T	C	0.83	0.0236	0.0022	3.12E-25
FG	rs11020124	11	92690661	T	C	0.72	-0.0599	0.0019	1.00E-200
FG	rs192701415	11	92979641	A	G	0.90	0.0201	0.0034	8.02E-09
FG	rs6489811	12	121893626	A	G	0.49	-0.0110	0.0018	3.27E-09
FG	rs11610045	12	133063768	A	G	0.45	0.0144	0.0019	3.26E-13
FG	rs2657879	12	56865338	A	G	0.80	-0.0119	0.0022	7.33E-09
FG	rs4760278	12	57771153	A	C	0.18	-0.0110	0.0020	3.33E-08
FG	rs6538804	12	97848910	C	G	0.62	0.0142	0.0019	9.41E-14
FG	rs11619319	13	28487599	A	G	0.77	-0.0173	0.0020	3.41E-20
FG	rs12888855	14	100830818	A	C	0.19	-0.0135	0.0020	6.02E-12
FG	rs35889227	14	90055468	T	G	0.62	-0.0130	0.0019	3.37E-10
FG	rs17270243	15	60862500	A	G	0.76	-0.0104	0.0021	3.62E-08
FG	rs7163757	15	62391608	T	C	0.43	-0.0217	0.0016	2.64E-36
FG	rs12898997	15	75090349	T	C	0.60	-0.0098	0.0017	4.64E-09
FG	rs7178572	15	77747190	A	G	0.32	-0.0121	0.0018	7.09E-10
FG	rs6598541	15	99271135	A	G	0.35	0.0114	0.0017	4.12E-12
FG	rs2238435	16	4014282	C	G	0.38	0.0112	0.0019	3.82E-09
FG	rs2302593	19	46196634	C	G	0.51	0.0106	0.0017	5.67E-10
FG	rs6113722	20	22557099	A	G	0.07	-0.0424	0.0044	7.66E-25
FG	rs17265513	20	39832628	T	C	0.80	-0.0158	0.0021	5.10E-14
FG	rs39713	22	30343186	T	C	0.06	-0.0169	0.0031	1.77E-08
FI	rs6674544	1	219628973	A	G	0.57	0.0177	0.0020	6.97E-21
FI	rs13389219	2	165528876	T	C	0.41	-0.0199	0.0019	5.84E-28
FI	rs2943646	2	227099534	A	G	0.38	-0.0250	0.0019	8.47E-39
FI	rs1260326	2	27730940	T	C	0.41	-0.0231	0.0019	8.42E-38
FI	rs5017305	2	630902	A	T	0.24	0.0137	0.0026	1.07E-08
FI	rs17036126	3	12287863	T	C	0.13	0.0209	0.0030	1.28E-10
FI	rs11708067	3	123065778	A	G	0.82	-0.0135	0.0023	1.30E-09
FI	rs35000407	3	12351521	T	G	0.88	0.0258	0.0028	1.50E-21
FI	rs62271373	3	150066540	A	T	0.06	0.0256	0.0048	1.60E-08
FI	rs10865959	3	49891002	C	G	0.30	0.0138	0.0022	1.99E-08

FI	rs17331151	3	52844534	T	C	0.11	-0.0162	0.0031	1.52E-08
FI	rs9884482	4	106081636	T	C	0.61	-0.0125	0.0019	2.88E-11
FI	rs11727676	4	145659064	T	C	0.92	-0.0203	0.0039	2.90E-08
FI	rs6855363	4	157670537	T	C	0.65	0.0125	0.0020	4.04E-08
FI	rs3775380	4	89739808	A	G	0.50	-0.0119	0.0018	1.48E-11
FI	rs10050393	5	157918946	T	C	0.54	0.0090	0.0019	4.84E-08
FI	rs4865796	5	53272664	A	G	0.71	0.0165	0.0020	7.33E-17
FI	rs459193	5	55806751	A	G	0.29	-0.0181	0.0021	1.12E-18
FI	rs1474696	6	127449246	A	G	0.52	-0.0147	0.0018	3.02E-16
FI	rs73013411	6	164126233	A	C	0.12	-0.0180	0.0032	2.08E-08
FI	rs116141873	6	34222201	T	G	0.03	0.0428	0.0059	1.42E-11
FI	rs2780215	6	34236973	A	G	0.96	0.0392	0.0063	1.06E-09
FI	rs6905288	6	43758873	A	G	0.60	0.0112	0.0019	7.75E-09
FI	rs972283	7	130466854	A	G	0.46	-0.0105	0.0019	1.09E-08
FI	rs2108349	7	50786663	A	G	0.69	-0.0115	0.0020	1.13E-08
FI	rs13258890	8	23615445	T	C	0.75	0.0128	0.0025	2.77E-08
FI	rs7012814	8	9173358	A	G	0.47	-0.0219	0.0019	8.34E-30
FI	rs75179845	9	136132954	T	C	0.92	-0.0216	0.0035	6.05E-11
FI	rs7903146	10	114758349	T	C	0.31	-0.0116	0.0021	1.24E-09
FI	rs118164457	10	89680631	T	C	0.96	-0.0345	0.0057	3.86E-10
FI	rs2845885	11	63869062	T	C	0.93	-0.0204	0.0039	1.18E-08
FI	rs860598	12	102898446	A	G	0.82	0.0177	0.0025	6.88E-12
FI	rs7133378	12	124409502	A	G	0.34	-0.0127	0.0020	6.00E-11
FI	rs6487237	12	21699928	A	C	0.79	0.0154	0.0026	4.68E-09
FI	rs1351394	12	66351826	T	C	0.47	-0.0111	0.0018	2.71E-09
FI	rs12454712	18	60845884	T	C	0.60	0.0142	0.0025	1.78E-09
FI	rs731839	19	33899065	A	G	0.66	-0.0121	0.0019	3.87E-11
FI	rs1206760	20	45582472	A	G	0.52	-0.0112	0.0019	8.82E-10
HbA1c	rs267738	1	150940625	T	G	0.80	0.0109	0.0016	1.14E-11
HbA1c	rs857725	1	158607935	T	G	0.72	-0.0208	0.0014	5.43E-55
HbA1c	rs7547793	1	203653544	A	C	0.12	-0.0118	0.0021	6.61E-09
HbA1c	rs340882	1	214145731	C	G	0.42	-0.0084	0.0013	1.48E-10
HbA1c	rs2375278	1	25529038	A	G	0.18	0.0112	0.0017	1.05E-11
HbA1c	rs1175549	1	3691727	A	C	0.79	0.0098	0.0015	7.13E-13
HbA1c	rs560887	2	169763148	T	C	0.31	-0.0307	0.0014	5.55E-122
HbA1c	rs13389076	2	169789512	A	G	0.03	0.0332	0.0038	3.04E-18
HbA1c	rs13419763	2	219134950	T	C	0.59	0.0080	0.0014	5.48E-09
HbA1c	rs12612492	2	24093756	T	C	0.15	0.0188	0.0019	1.88E-26
HbA1c	rs1367173	2	43449385	T	C	0.11	-0.0152	0.0020	1.66E-14
HbA1c	rs79403657	2	48114094	C	G	0.82	-0.0090	0.0017	2.03E-08
HbA1c	rs10169706	2	5791194	T	C	0.04	0.0260	0.0046	1.48E-08
HbA1c	rs12491937	3	12268244	A	G	0.56	0.0090	0.0013	1.42E-13
HbA1c	rs11719201	3	123068744	T	C	0.18	-0.0129	0.0015	2.43E-18
HbA1c	rs6804915	3	170627909	A	C	0.29	-0.0108	0.0014	2.76E-16
HbA1c	rs13089972	3	171798694	A	T	0.58	0.0111	0.0014	1.87E-15
HbA1c	rs9818758	3	49382925	A	G	0.20	0.0131	0.0017	1.49E-13
HbA1c	rs6798941	3	52893465	T	C	0.32	0.0086	0.0015	1.49E-08
HbA1c	rs13134327	4	144659795	A	G	0.33	0.0144	0.0014	2.81E-26
HbA1c	rs6877043	5	154048367	T	C	0.64	0.0085	0.0014	1.99E-10
HbA1c	rs9376090	6	135411228	T	C	0.73	0.0247	0.0014	1.90E-62

HbA1c	rs10946402	6	20715826	T	G	0.83	-0.0101	0.0016	1.12E-10
HbA1c	rs1800562	6	26093141	A	G	0.05	-0.0383	0.0027	2.33E-50
HbA1c	rs204995	6	32154285	A	G	0.78	-0.0098	0.0018	1.93E-09
HbA1c	rs3778321	6	7250270	A	G	0.18	-0.0106	0.0016	4.18E-11
HbA1c	rs4727979	7	123429697	A	C	0.91	0.0121	0.0024	4.61E-08
HbA1c	rs10231021	7	15060429	A	T	0.49	0.0089	0.0013	8.69E-14
HbA1c	rs2908277	7	44183433	A	G	0.12	0.0166	0.0020	1.29E-18
HbA1c	rs2971670	7	44226101	T	C	0.18	0.0316	0.0017	5.10E-88
HbA1c	rs13234131	7	73025975	A	G	0.88	-0.0113	0.0020	2.06E-09
HbA1c	rs11558471	8	118185733	A	G	0.71	0.0151	0.0014	3.38E-25
HbA1c	rs2001846	8	126478450	T	C	0.47	-0.0069	0.0013	8.58E-10
HbA1c	rs6474359	8	41549194	T	C	0.98	0.0427	0.0038	1.91E-33
HbA1c	rs4737009	8	41630405	A	G	0.26	0.0228	0.0015	8.29E-56
HbA1c	rs7042939	9	110511408	A	G	0.42	0.0102	0.0013	1.50E-15
HbA1c	rs651007	9	136153875	T	C	0.22	0.0108	0.0015	3.28E-15
HbA1c	rs3829109	9	139256766	A	G	0.28	-0.0086	0.0015	2.68E-08
HbA1c	rs10811661	9	22134094	T	C	0.84	0.0128	0.0017	1.74E-14
HbA1c	rs7861647	9	79977386	T	C	0.19	0.0128	0.0016	4.50E-14
HbA1c	rs61750929	9	91495135	T	C	0.04	-0.0284	0.0029	9.49E-24
HbA1c	rs7903146	10	114758349	T	C	0.31	0.0133	0.0014	1.04E-22
HbA1c	rs11257655	10	12307894	T	C	0.24	0.0110	0.0016	1.91E-13
HbA1c	rs2102339	10	71015389	T	C	0.33	-0.0087	0.0014	3.42E-10
HbA1c	rs16926246	10	71093392	T	C	0.14	-0.0727	0.0021	1.00E-200
HbA1c	rs7127313	11	100508897	T	C	0.34	0.0066	0.0013	4.85E-08
HbA1c	rs608793	11	118986659	T	C	0.48	0.0065	0.0013	4.55E-08
HbA1c	rs4980325	11	234451	T	G	0.53	0.0108	0.0014	4.70E-14
HbA1c	rs11039154	11	47278502	T	C	0.28	-0.0087	0.0014	3.11E-09
HbA1c	rs174559	11	61581656	A	G	0.29	-0.0106	0.0014	3.31E-13
HbA1c	rs10830963	11	92708710	C	G	0.71	-0.0197	0.0015	1.54E-36
HbA1c	rs360147	11	9790817	T	C	0.26	-0.0086	0.0015	2.08E-09
HbA1c	rs10774624	12	111833788	A	G	0.53	0.0093	0.0013	4.17E-14
HbA1c	rs117233107	12	4328521	A	G	0.02	-0.0470	0.0072	8.45E-11
HbA1c	rs4760682	12	48512285	A	C	0.82	0.0164	0.0018	3.20E-20
HbA1c	rs76533333	13	113352916	A	G	0.91	-0.0265	0.0025	2.81E-29
HbA1c	rs1278769	13	113536627	A	G	0.23	-0.0091	0.0015	5.52E-12
HbA1c	rs1535464	14	100793431	A	G	0.21	-0.0086	0.0017	1.11E-08
HbA1c	rs151165	14	65272626	A	T	0.40	0.0079	0.0014	2.04E-09
HbA1c	rs10151436	14	73616095	A	T	0.89	0.0130	0.0021	3.85E-11
HbA1c	rs452306	15	65822777	T	C	0.63	-0.0098	0.0014	5.51E-13
HbA1c	rs11643024	16	11443183	A	G	0.30	0.0084	0.0015	7.98E-10
HbA1c	rs7190771	16	28590030	A	G	0.33	0.0085	0.0013	6.02E-11
HbA1c	rs11248914	16	293562	T	C	0.70	0.0114	0.0014	1.42E-14
HbA1c	rs7198799	16	68818390	T	C	0.28	0.0083	0.0014	4.76E-09
HbA1c	rs837763	16	88853729	T	C	0.58	0.0176	0.0013	5.20E-38
HbA1c	rs9914988	17	27183104	A	G	0.80	0.0125	0.0016	4.66E-17
HbA1c	rs2748427	17	76121864	A	G	0.80	-0.0307	0.0022	9.82E-49
HbA1c	rs9909940	17	80689036	T	C	0.32	0.0322	0.0014	1.43E-116
HbA1c	rs28671200	18	43774444	T	G	0.65	0.0086	0.0017	1.56E-08
HbA1c	rs17533945	19	17257802	T	C	0.58	-0.0128	0.0014	1.62E-23
HbA1c	rs10405535	19	33072085	A	G	0.29	0.0122	0.0016	6.47E-14

HbA1c	rs737092	20	55990405	T	C	0.50	-0.0073	0.0013	7.57E-09
HbA1c	rs855791	22	37462936	A	G	0.40	0.0188	0.0013	1.34E-56
HbA1c	rs8138197	22	43114551	A	G	0.49	-0.0073	0.0014	3.54E-08

Chr, chromosome; EA, effect allele; EAF, effect allele frequency; SE, standard error.

Supplementary Table 2. Outcome data information

Outcome	Data source	Population	Cases	Controls
**Atherosclerotic outcome**				
Coronary artery disease	CARDIoGRAMplusC4D plus UKBB[a]	Mixed	122,733	424,528
	FinnGen[b]	European	21,012	197,780
Angina	FinnGen[b]	European	18,168	187,840
	UKBB[c]	European	25,353	342,189
Coronary atherosclerosis	FinnGen[b]	European	23,363	187,840
	UKBB[c]	European	36,926	330,616
Coronary revascularization	FinnGen[b]	European	12,271	187,840
Ischemic stroke	MEGASTROKE consortium[d]	European	34,217	406,111
	FinnGen[b]	European	10,551	202,223
	UKBB[c]	European	7306	360,236
Large artery stroke	MEGASTROKE consortium[d]	European	4373	406,111
Small vessel stroke	MEGASTROKE consortium[d]	European	5386	192,662
Cardioembolic stroke	MEGASTROKE consortium[d]	European	7193	406,111
Transient ischemic attack	FinnGen[b]	European	8835	202,223
	UKBB[c]	European	5162	362,380
Aortic aneurysm	FinnGen[b]	European	2825	206,541
	UKBB[c]	European	3157	364,385
Peripheral artery disease	FinnGen[b]	European	7098	206,541
	UKBB[c]	European	4934	362,608
Chronic kidney disease	CKD Gen consortium[e]	European	64,164	625,219
	FinnGen[b]	European	3902	212,841
**Thrombotic outcome**				
Subarachnoid hemorrhage	Consortium (Bakker et al)[f]	European	5140	71,952
	FinnGen[b]	European	1338	201,230
Venous thromboembolism	FinnGen[b]	European	9176	209,616
	UKBB[c]	European	17,392	350,150
Deep venous thrombosis	FinnGen[b]	European	4576	190,028
	UKBB[c]	European	10,690	356,852
Pulmonary embolism	FinnGen[b]	European	4185	214,228
	UKBB[c]	European	8474	359,068

UKBB, UK Biobank. Age, sex, and ten genetic principal components were adjusted in UKBB genome-wide association analysis. Age, sex, the first ten genetic principal components, and genotyping batch were adjusted in FinnGen genome-wide association analysis. Age and sex were adjusted in MEGASTROKE consortium and CKD Gen consortium genome-wide association analysis.

[a] van der Harst P, Verweij N: Identification of 64 Novel Genetic Loci Provides an Expanded View on the Genetic Architecture of Coronary Artery Disease. Circ Res 2018;122:433-443.

[b] The FinnGen consortium. R5 release of genome-wide association analysis results. https://finngen.gitbook.io/documentation/. Assessed on 6, July, 2021

[c] Sudlow C, Gallacher J, Allen N, Beral V, Burton P, Danesh J, et al. UK biobank: an open access resource for identifying the causes of a wide range of complex diseases of middle and old age. PLoS Med. 2015;12(3):e1001779.
[d] Malik R, Chauhan G, Traylor M, Sargurupremraj M, Okada Y, Mishra A, et al: Multiancestry genome-wide association study of 520,000 subjects identifies 32 loci associated with stroke and stroke subtypes. Nat Genet 2018;50:524-537
[e] Wuttke M, Li Y, Li M, Sieber KB, Feitosa MF, Gorski M, et al: A catalog of genetic loci associated with kidney function from analyses of a million individuals. Nat Genet 2019;51:957-972
[f] Bakker MK, van der Spek RAA, van Rheenen W, Morel S, Bourcier R, Hostettler IC, et al: Genome-wide association study of intracranial aneurysms identifies 17 risk loci and genetic overlap with clinical risk factors. Nat Genet 2020;52:1303-1313

Supplementary Table 3. Diagnostic information in FinnGen and UK Biobank

Outcome	Data source	ICD-8 diagnosis	ICD-9 diagnosis	ICD-10 diagnosis	Surgery code	Self-report†
**Atherosclerotic outcome**						
Coronary artery disease	FinnGen	410|4110	410|4110	I20.0, I21, I22	-	-
Angina	FinnGen	413	413|411[0-1]	I20	-	-
	UKBB	413	413|411[0-1]	I20	-	-
Coronary atherosclerosis	FinnGen	414	414|9960A	I24, I25, T82.2, Z95.1	-	-
	UKBB	414	414|9960A	I24, I25, T82.2, Z95.1	-	-
Coronary revascularization	FinnGen	-	-	-	^FNF|^FNG|^TFN40|^FN1AT|^FN1BT|^FN1YT, ^FNA|^FNB|^FNC|^FND|^FNE, ^82$\|^83$|^84$,^11$|^25$\|^111$|^112$\|^113$|^119$, ^AN2\|^AN3\|^AN4\|^ANA\|^ANB,^AA1\|^AA2\|^AA3\|^AAX, ^5311$|^5312$\|^5313$|^5314$\|^5315$	-
Ischemic stroke	FinnGen	433|434|436	4330A|4331A|4339A|4340A|4341A|4349A|436	I63, I64	-	-
	UKBB	-	434, 436	I63, I64	-	20002
Transient ischemic attack	FinnGen	435	435	G45	-	-
	UKBB	-	435	G45	-	20002
Aortic aneurysm	FinnGen	0930|441	441[1-9]|0930A	I71.1, I71.2, I71.3, I71.4, I71.5, I71.6, I71.8, I71.9	-	-
	UKBB	-	441.3, 441.4, 441.1, 441.2	I71.3, I71.4, I71.1, I71.2	L19.4, L19.5	20002
Peripheral artery disease	FinnGen	25006|4402|4439	4402|4439	E10.5, E10.5+I79.2, E11.5, E11.5+I79.2, E12.5, E13.5, E14.5, I70.2, I73.9	-	-
	UKBB	-	443.8, 443.9	I73.8, I73.9	-	20002
Chronic kidney disease	FinnGen	-	585	N18	-	-
**Thrombotic outcome**						
Subarachnoid hemorrhage	FinnGen	430	430	I60	-	-
Venous thromboembolism	FinnGen	450|451|671|6739	415|451|6713|6714|6732	I26, I80, O87.1, O88.2	-	-
	UKBB	-	415.1, 451.1, 452, 453.0, 453.4, 453.9	I26, I80.1, I80.2, I81, I82.0	L90.2	20002, 6152
Deep venous thrombosis	FinnGen	4510	4511A|4510A|4512A	I80.20#, I80.29#, I80.3#	-	-
	UKBB	-	451.1	I80.2	L90.2	20002, 6152
Pulmonary embolism	FinnGen	450	415	I26		
	UKBB	-	415.1	I26	-	20002, 6152

Supplementary Table 4. Strength of genetic instruments

Cardiovascular disease	Data source	Sample size	FG	2hGlu	FI	HbA1c
**Atherosclerotic outcome**						
Coronary artery disease	CARDIoGRAMplusC4D plus UKBB	547261	280	315	53	316
	FinnGen	218792	112	126	21	126
Angina	FinnGen	206008	105	119	20	119
	UKBB	367542	188	212	36	213
Coronary atherosclerosis	FinnGen	211203	108	122	20	122
	UKBB	367542	188	212	36	213
Coronary revascularization	FinnGen	200111	102	115	19	116
Ischemic stroke	MEGASTROKE consortium	440328	225	254	43	255
	FinnGen	212774	109	123	21	123
	UKBB	367542	188	212	36	213
Large artery stroke	MEGASTROKE consortium	410484	210	236	40	237
Small vessel stroke	MEGASTROKE consortium	198048	101	114	19	114
Cardioembolic stroke	MEGASTROKE consortium	413304	211	238	40	239
Transient ischemic attack	FinnGen	211058	108	122	20	122
	UKBB	367542	188	212	36	213
Aortic aneurysm	FinnGen	209366	107	121	20	121
	UKBB	367542	188	212	36	213
Peripheral artery disease	FinnGen	213639	109	123	21	124
	UKBB	367542	188	212	36	213
Chronic kidney disease	CKD Gen consortium	689383	353	397	67	399
	FinnGen	216743	111	125	21	125
**Thrombotic outcome**						
Subarachnoid hemorrhage	Consortium (Bakker et al)	77092	39	44	7	45
	FinnGen	202568	104	117	20	117
Venous thromboembolism	FinnGen	218792	112	126	21	126
	UKBB	367542	188	212	36	213
Deep venous thrombosis	FinnGen	194604	100	112	19	112
	UKBB	367542	188	212	36	213
Pulmonary embolism	FinnGen	218413	112	126	21	126
	UKBB	367542	188	212	36	213

2hGlu, 2-h glucose after an oral glucose challenge; FG, fasting glucose; FI, fasting insulin; HbA1c, glycated hemoglobin; UKBB, UK Biobank.

Supplementary Table 5. False discovery rate correction

Trait	Outcome	Original P value	Benjamini-Hochberg Adjusted P value	Significant using an FDR of 0.05?
FG	Coronary atherosclerosis	6.78E-06	1.09E-04	Yes
FG	Peripheral artery disease	9.22E-04	7.38E-03	Yes
FG	Angina	3.84E-03	2.05E-02	Yes
FG	Coronary artery disease	8.94E-03	3.51E-02	Yes
FG	Large artery stroke	1.10E-02	3.51E-02	Yes
FG	Ischemic stroke	2.18E-02	5.81E-02	Suggestive
FG	Coronary revascularization	3.78E-02	8.64E-02	Suggestive
FG	Subarachnoid hemorrhage	8.86E-02	1.77E-01	No
FG	Chronic kidney disease	1.08E-01	1.92E-01	No
FG	Aortic aneurysm	2.17E-01	3.47E-01	No
FG	Small vessel stroke	3.01E-01	4.26E-01	No
FG	Cardioembolic stroke	3.19E-01	4.26E-01	No
FG	Transient ischemic attack	9.99E-01	9.99E-01	No
FG	Venous thromboembolism	9.87E-01	9.99E-01	No
FG	Deep venous thrombosis	8.30E-01	9.99E-01	No
FG	Pulmonary embolism	9.66E-01	9.99E-01	No
2hGlu	Coronary artery disease	1.84E-03	9.82E-03	Yes
2hGlu	Peripheral artery disease	1.78E-03	9.82E-03	Yes
2hGlu	Pulmonary embolism	1.37E-03	9.82E-03	Yes
2hGlu	Small vessel stroke	2.94E-03	1.17E-02	Yes
2hGlu	Angina	7.45E-03	2.38E-02	Yes
2hGlu	Large artery stroke	1.21E-02	3.23E-02	Yes
2hGlu	Coronary atherosclerosis	3.50E-02	7.00E-02	Suggestive
2hGlu	Aortic aneurysm	3.08E-02	7.00E-02	Suggestive
2hGlu	Venous thromboembolism	3.94E-02	7.01E-02	Suggestive
2hGlu	Ischemic stroke	7.03E-02	1.03E-01	No
2hGlu	Subarachnoid hemorrhage	7.08E-02	1.03E-01	No
2hGlu	Coronary revascularization	8.10E-02	1.08E-01	No
2hGlu	Cardioembolic stroke	2.24E-01	2.76E-01	No
2hGlu	Deep venous thrombosis	6.53E-01	7.47E-01	No
2hGlu	Transient ischemic attack	8.68E-01	8.71E-01	No
2hGlu	Chronic kidney disease	8.71E-01	8.71E-01	No
FI	Coronary atherosclerosis	5.14E-07	8.22E-06	Yes
FI	Coronary artery disease	1.80E-06	1.44E-05	Yes
FI	Angina	6.14E-05	3.27E-04	Yes
FI	Coronary revascularization	7.07E-04	2.83E-03	Yes
FI	Peripheral artery disease	2.76E-03	8.83E-03	Yes
FI	Chronic kidney disease	4.83E-03	1.29E-02	Yes
FI	Ischemic stroke	1.56E-02	3.56E-02	Yes
FI	Small vessel stroke	3.93E-02	7.87E-02	Suggestive
FI	Transient ischemic attack	1.05E-01	1.86E-01	No

FI	Aortic aneurysm	1.54E-01	2.47E-01	No
FI	Large artery stroke	4.31E-01	6.27E-01	No
FI	Cardioembolic stroke	4.99E-01	6.65E-01	No
FI	Subarachnoid hemorrhage	7.85E-01	8.53E-01	No
FI	Venous thromboembolism	8.53E-01	8.53E-01	No
FI	Deep venous thrombosis	8.28E-01	8.53E-01	No
FI	Pulmonary embolism	8.12E-01	8.53E-01	No
HbA1c	Coronary atherosclerosis	3.00E-03	4.80E-02	Yes
HbA1c	Coronary artery disease	1.60E-02	8.56E-02	Suggestive
HbA1c	Angina	1.29E-02	8.56E-02	Suggestive
HbA1c	Large artery stroke	1.12E-01	3.58E-01	No
HbA1c	Peripheral artery disease	1.00E-01	3.58E-01	No
HbA1c	Aortic aneurysm	1.51E-01	4.04E-01	No
HbA1c	Coronary revascularization	3.28E-01	5.84E-01	No
HbA1c	Small vessel stroke	2.65E-01	5.84E-01	No
HbA1c	Cardioembolic stroke	3.00E-01	5.84E-01	No
HbA1c	Subarachnoid hemorrhage	4.73E-01	6.42E-01	No
HbA1c	Chronic kidney disease	4.74E-01	6.42E-01	No
HbA1c	Deep venous thrombosis	4.81E-01	6.42E-01	No
HbA1c	Pulmonary embolism	5.81E-01	7.15E-01	No
HbA1c	Ischemic stroke	8.86E-01	9.45E-01	No
HbA1c	Transient ischemic attack	8.33E-01	9.45E-01	No
HbA1c	Venous thromboembolism	9.96E-01	9.96E-01	No

Supplementary Table 6. Associations for HbA1c after adjusting for genetically predicted red blood cell distribution width

Outcome	OR	95% CI	P value	P_FDR
Coronary artery disease	1.28	1.04, 1.58	0.021	0.076
Angina	1.31	1.05, 1.64	0.015	0.076
Coronary atherosclerosis	1.42	1.13, 1.79	0.003	0.048
Coronary revascularization	1.38	0.82, 2.33	0.223	0.398
Ischemic stroke	0.99	0.81, 1.20	0.886	0.886
Large artery stroke	1.47	0.76, 2.86	0.256	0.410
Small vessel stroke	1.36	0.83, 2.21	0.224	0.398
Cardioembolic stroke	0.81	0.53, 1.22	0.306	0.445
Transient ischemic attack	0.94	0.71, 1.25	0.672	0.717
Aortic aneurysm	0.66	0.44, 1.01	0.056	0.129
Peripheral artery disease	1.46	1.04, 2.05	0.028	0.076
Chronic kidney disease	1.06	0.81, 1.38	0.664	0.717
Subarachnoid hemorrhage	0.82	0.49, 1.39	0.463	0.570
Venous thromboembolism	0.78	0.62, 0.97	0.028	0.076
Deep venous thrombosis	0.68	0.51, 0.90	0.007	0.054
Pulmonary embolism	0.88	0.68, 1.16	0.375	0.500

CI, confidence interval; OR, odds ratio.

Supplementary Table 7. Associations of genetically predicted glycemic traits with atherosclerotic and thrombotic outcomes in sensitivity analysis

Outcome	Source	SNPs used	Cochran's Q value	$P_{intercept}$	Weighted median			MR-Egger			Contamination mix		
					OR	95% CI	*P*	OR	95% CI	*P*	OR	95% CI	*P*
**Fasting glucose**													
Coronary artery disease	Consortium	69	219	0.540	1.20	1.07, 1.33	0.001	0.79	0.52, 1.19	0.281	1.39	1.28, 1.52	1.79E-07
	FinnGen	70	108	0.065	1.13	0.91, 1.41	0.278	0.93	0.67, 1.28	0.648	1.14	0.97, 1.35	0.122
Angina	UKBB	70	111	0.351	1.12	0.84, 1.48	0.443	0.99	0.65, 1.50	0.949	1.14	0.90, 1.45	0.349
	FinnGen	70	155	0.743	1.36	1.11, 1.66	0.003	1.20	0.85, 1.69	0.295	1.45	1.27, 1.67	2.40E-05
Coronary atherosclerosis	UKBB	70	99	0.104	1.28	1.01, 1.62	0.045	1.01	0.71, 1.44	0.954	1.27	1.05, 1.52	0.016
	FinnGen	70	182	0.996	1.40	1.18, 1.68	0.000	1.35	0.98, 1.85	0.069	1.49	1.32, 1.72	0.001
Coronary revascularization	FinnGen	70	117	0.130	1.32	0.94, 1.85	0.107	0.95	0.57, 1.58	0.839	1.20	0.92, 1.54	0.164
Ischemic stroke	Consortium	70	133	0.027	0.98	0.79, 1.20	0.820	0.88	0.63, 1.23	0.457	1.06	0.90, 1.25	0.558
	UKBB	70	92	0.254	0.94	0.68, 1.30	0.707	0.87	0.57, 1.31	0.494	1.00	0.79, 1.23	0.999
	FinnGen	70	82	0.139	1.22	0.88, 1.68	0.230	1.50	0.97, 2.31	0.075	1.13	0.86, 1.40	0.371
Large artery stroke	Consortium	70	121	0.337	1.48	0.87, 2.53	0.150	1.23	0.56, 2.70	0.609	1.26	0.53, 2.59	0.344
Small vessel stroke	Consortium	70	79	0.029	0.64	0.40, 1.02	0.060	0.67	0.38, 1.20	0.184	3.06	1.65, 4.85	0.014
Cardioembolic stroke	Consortium	70	89	0.595	0.96	0.65, 1.44	0.858	1.02	0.60, 1.73	0.950	1.04	0.75, 1.42	0.896
Transient ischemic attack	UKBB	70	74	0.014	0.80	0.58, 1.10	0.162	0.67	0.45, 0.98	0.042	0.86	0.66, 1.08	0.202
	FinnGen	70	80	0.060	0.87	0.57, 1.32	0.506	0.64	0.39, 1.06	0.088	0.81	0.53, 1.19	0.246
Aortic aneurysm	UKBB	70	90	0.909	0.74	0.43, 1.30	0.297	0.68	0.32, 1.48	0.336	0.67	0.44, 1.04	0.077
	FinnGen	70	95	0.923	1.18	0.73, 1.92	0.493	0.95	0.46, 1.95	0.881	0.98	0.66, 1.52	0.935
Peripheral artery disease	UKBB	70	133	0.564	1.50	1.05, 2.14	0.028	1.35	0.73, 2.49	0.337	1.43	1.08, 1.95	0.025
	FinnGen	70	82	0.789	1.26	0.84, 1.88	0.256	1.24	0.72, 2.12	0.436	1.28	0.90, 1.72	0.188
Chronic kidney disease	Consortium	70	149	0.218	0.98	0.79, 1.22	0.847	1.13	0.80, 1.58	0.496	1.04	0.80, 1.20	0.521
	FinnGen	70	69	0.996	1.43	0.88, 2.32	0.150	1.29	0.73, 2.27	0.389	1.13	0.82, 1.86	0.447
Subarachnoid hemorrhage	Consortium	55	99	0.278	0.82	0.48, 1.39	0.457	0.91	0.41, 2.02	0.812	0.74	0.33, 1.17	0.210
	FinnGen	70	67	0.380	0.79	0.32, 1.95	0.609	0.66	0.26, 1.69	0.387	0.46	0.16, 2.12	NA
Venous thromboembolism	UKBB	70	371	0.267	0.75	0.55, 1.04	0.083	0.62	0.26, 1.49	0.293	0.78	0.58, 0.99	0.048
	FinnGen	70	617	0.370	0.85	0.67, 1.09	0.206	0.76	0.34, 1.68	0.494	0.74	0.62, 0.95	0.015
Deep venous thrombosis	UKBB	70	284	0.265	0.65	0.42, 1.01	0.055	0.58	0.20, 1.69	0.326	0.68	0.45, 1.02	0.065
	FinnGen	70	445	0.571	1.03	0.77, 1.38	0.841	0.87	0.37, 2.05	0.751	0.91	0.76, 1.14	0.471
Pulmonary embolism	UKBB	70	175	0.249	0.79	0.51, 1.24	0.305	0.61	0.26, 1.44	0.264	0.84	0.51, 1.30	0.428
	FinnGen	70	414	0.281	0.80	0.59, 1.10	0.167	0.68	0.27, 1.71	0.417	0.77	0.59, 1.00	0.059
**2hGlu**													
Coronary artery disease	Consortium	12	79	0.083	1.44	1.23, 1.68	5.03E-06	1.20	0.89, 1.62	0.234	1.22	1.14, 1.34	5.26E-05
	FinnGen	13	31	0.040	1.07	0.91, 1.26	0.411	0.72	0.49, 1.05	0.113	1.23	0.96, 1.45	0.011
Angina	UKBB	13	23	0.027	1.17	0.97, 1.41	0.109	0.70	0.48, 1.04	0.108	1.22	0.97, 1.54	0.017
	FinnGen	14	64	0.070	1.26	1.09, 1.45	0.002	0.81	0.52, 1.28	0.385	1.45	1.26, 1.65	5.90E-09
Coronary atherosclerosis	UKBB	13	32	0.086	1.15	0.95, 1.39	0.146	0.72	0.45, 1.15	0.193	1.27	0.95, 1.54	0.015
	FinnGen	14	71	0.058	1.25	1.11, 1.41	0.000	0.79	0.53, 1.18	0.270	1.30	1.20, 1.42	0.000
Coronary revascularization	FinnGen	13	36	0.143	1.18	0.93, 1.50	0.173	0.76	0.39, 1.49	0.439	1.14	0.76, 1.92	0.717
Ischemic stroke	Consortium	13	45	0.453	1.07	0.93, 1.24	0.337	0.98	0.60, 1.57	0.923	1.08	0.90, 1.21	0.117
	UKBB	13	13	0.372	1.02	0.84, 1.23	0.845	0.91	0.62, 1.35	0.665	1.08	0.90, 1.25	0.501
	FinnGen	14	22	0.861	1.01	0.82, 1.23	0.947	0.98	0.57, 1.68	0.934	0.98	0.84, 1.17	0.802
Large artery stroke	Consortium	13	30	0.519	1.65	1.17, 2.34	0.005	1.15	0.41, 3.21	0.790	1.30	1.01, 2.05	0.034
Small vessel stroke	Consortium	13	16	0.403	1.67	1.26, 2.23	4.34E-04	1.92	0.98, 3.74	0.083	1.68	1.30, 2.10	1.01E-04
Cardioembolic stroke	Consortium	13	34	0.356	1.05	0.82, 1.34	0.696	0.83	0.37, 1.88	0.664	1.05	0.88, 1.26	0.580

Transient ischemic attack	UKBB	13	13	0.091	1.00	0.81, 1.23	0.997	0.72	0.48, 1.08	0.143	1.03	0.88, 1.22	0.675
	FinnGen	14	16	0.804	1.05	0.82, 1.34	0.684	0.92	0.53, 1.58	0.756	0.98	0.79, 1.30	0.781
Aortic aneurysm	UKBB	13	16	0.474	0.74	0.50, 1.10	0.133	0.97	0.43, 2.22	0.949	0.66	0.48, 1.14	0.246
	FinnGen	14	15	0.665	1.01	0.73, 1.38	0.973	0.76	0.39, 1.51	0.449	1.13	0.71, 1.45	0.472
Peripheral artery disease	UKBB	13	25	0.151	1.33	1.01, 1.75	0.042	0.89	0.47, 1.67	0.720	1.72	1.02, 2.64	0.009
	FinnGen	14	36	0.348	1.21	0.91, 1.60	0.193	0.86	0.38, 1.95	0.717	1.43	0.89, 1.79	0.193
Chronic kidney disease	Consortium	13	34	0.545	1.00	0.88, 1.12	0.954	1.10	0.75, 1.61	0.629	1.02	0.90, 1.14	0.537
	FinnGen	13	10	0.999	1.00	0.75, 1.32	0.977	1.06	0.59, 1.91	0.846	1.00	0.80, 1.23	0.999
Subarachnoid hemorrhage	Consortium	9	11	0.104	0.94	0.66, 1.32	0.704	0.56	0.27, 1.16	0.164	0.98	0.75, 1.27	0.858
	FinnGen	13	13	0.671	1.76	1.05, 2.92	0.031	1.24	0.43, 3.62	0.697	1.40	0.96, 2.59	0.072
Venous thromboembolism	UKBB	13	15	0.406	0.82	0.67, 1.00	0.053	0.70	0.45, 1.09	0.139	0.84	0.70, 1.03	0.034
	FinnGen	14	388	0.526	0.92	0.79, 1.07	0.255	0.66	0.15, 2.91	0.592	0.89	0.77, 1.02	0.005
Deep venous thrombosis	UKBB	13	15	0.644	0.84	0.63, 1.11	0.224	0.81	0.43, 1.54	0.537	1.00	0.82, 1.21	0.999
	FinnGen	14	274	0.478	0.89	0.75, 1.06	0.196	0.61	0.13, 2.91	0.544	0.85	0.75, 1.01	0.020
Pulmonary embolism	UKBB	13	10	0.809	0.72	0.54, 0.95	0.019	0.77	0.43, 1.35	0.374	0.72	0.58, 0.88	0.002
	FinnGen	14	250	0.635	0.90	0.73, 1.10	0.313	0.73	0.13, 3.97	0.718	0.90	0.74, 1.07	0.094
**Fasting insulin**													
Coronary artery disease	Consortium	37	170	0.356	1.97	1.48, 2.63	3.44E-06	1.07	0.35, 3.29	0.903	2.72	2.05, 3.46	3.23E-09
	FinnGen	38	81	0.354	1.68	1.08, 2.60	0.020	3.50	1.03, 11.9	0.052	2.03	1.20, 3.49	0.009
Angina	UKBB	38	99	0.614	2.04	1.15, 3.63	0.015	3.54	0.65, 19.4	0.154	5.05	0.70, 9.03	0.119
	FinnGen	38	107	0.675	2.43	1.68, 3.51	2.17E-06	1.32	0.44, 3.95	0.622	3.00	2.34, 4.10	3.97E-07
Coronary atherosclerosis	UKBB	38	75	0.564	1.90	1.19, 3.04	0.007	3.34	0.87, 12.8	0.087	1.79	1.25, 2.61	0.015
	FinnGen	38	113	0.710	1.57	1.13, 2.17	0.006	1.45	0.55, 3.79	0.455	2.44	1.77, 3.42	3.36E-06
Coronary revascularization	FinnGen	38	95	0.903	2.65	1.38, 5.10	0.004	2.61	0.35, 19.7	0.358	1.72	0.95, 17.29	0.073
Ischemic stroke	Consortium	37	72	0.957	1.21	0.85, 1.72	0.294	1.19	0.41, 3.46	0.746	1.31	1.01, 1.79	0.048
	UKBB	38	49	0.133	1.59	0.94, 2.69	0.082	4.06	1.14, 14.4	0.038	1.52	0.96, 2.56	0.069
	FinnGen	38	50	0.317	1.21	0.67, 2.19	0.524	2.30	0.61, 8.68	0.225	1.00	0.61, 2.64	0.999
Large artery stroke	Consortium	37	77	0.758	1.50	0.60, 3.70	0.384	0.94	0.06, 14.0	0.965	2.97	0.66, 25.79	0.125
Small vessel stroke	Consortium	37	76	0.515	4.10	1.74, 9.65	0.001	1.03	0.08, 12.9	0.981	13.07	3.71, 37.34	4.78E-04
Cardioembolic stroke	Consortium	37	38	0.539	0.69	0.35, 1.37	0.290	1.33	0.30, 5.89	0.711	0.55	0.23, 1.23	0.068
Transient ischemic attack	UKBB	38	39	0.726	1.34	0.75, 2.40	0.318	1.08	0.30, 3.81	0.908	1.21	0.67, 2.32	0.544
	FinnGen	38	44	0.603	1.11	0.57, 2.18	0.761	1.77	0.40, 7.85	0.459	1.23	0.62, 4.10	0.698
Aortic aneurysm	UKBB	38	64	0.635	1.65	0.61, 4.51	0.327	3.07	0.19, 49.2	0.434	5.93	0.23, 29.37	0.190
	FinnGen	38	63	0.186	1.20	0.50, 2.87	0.685	6.08	0.66, 56.3	0.121	0.31	0.08, 1.36	NA
Peripheral artery disease	UKBB	38	74	0.984	2.75	1.36, 5.55	0.005	2.37	0.33, 17.0	0.398	4.85	1.43, 10.28	0.005
	FinnGen	38	60	0.899	1.22	0.61, 2.45	0.573	1.71	0.29, 10.1	0.557	1.62	0.59, 3.63	0.336
Chronic kidney disease	Consortium	37	78	0.784	1.11	0.78, 1.60	0.556	0.68	0.25, 1.90	0.472	1.31	0.99, 1.67	0.063
	FinnGen	38	33	0.658	1.53	0.69, 3.38	0.295	1.51	0.26, 8.83	0.649	1.77	0.71, 3.97	0.204
Subarachnoid hemorrhage	Consortium	25	47	0.851	1.33	0.39, 4.55	0.651	0.69	0.02, 22.0	0.833	0.08	0.03, 58.56	0.073
	FinnGen	38	34	0.144	1.17	0.31, 4.44	0.822	0.15	0.01, 2.78	0.209	4.14	0.98, 50.91	0.054
Venous thromboembolism	UKBB	38	112	0.127	0.86	0.48, 1.55	0.617	4.30	0.57, 32.4	0.166	0.56	0.24, 2.03	0.386
	FinnGen	38	189	0.243	0.97	0.66, 1.42	0.870	2.57	0.48, 13.7	0.279	0.96	0.71, 1.51	0.789
Deep venous thrombosis	UKBB	38	109	0.259	0.48	0.22, 1.04	0.063	4.20	0.25, 70.2	0.325	0.68	0.15, 1.39	0.317
	FinnGen	38	145	0.419	1.14	0.71, 1.82	0.584	2.42	0.37, 15.7	0.360	1.00	0.68, 1.70	0.999
Pulmonary embolism	UKBB	38	79	0.010	1.17	0.52, 2.62	0.711	20.03	2.04, 196.8	0.014	1.06	0.52, 2.77	0.836
	FinnGen	38	91	0.220	1.08	0.64, 1.84	0.771	2.49	0.48, 12.89	0.285	0.72	0.50, 1.20	0.218
**HbA1c**													
Coronary artery disease	Consortium	74	321	0.936	1.35	1.09, 1.67	0.006	1.38	0.88, 2.18	0.164	1.58	1.38, 1.90	0.001
	FinnGen	75	150	0.390	1.29	0.93, 1.78	0.129	1.39	0.80, 2.41	0.246	1.23	0.93, 1.65	0.133

Angina	UKBB	75	118	0.445	0.99	0.67, 1.46	0.942	1.30	0.70, 2.38	0.408	1.04	0.78, 1.43	0.739
	FinnGen	75	179	0.580	1.39	1.08, 1.79	0.009	1.60	1.02, 2.52	0.045	1.54	1.27, 2.03	3.87E-05
Coronary atherosclerosis	UKBB	75	167	0.227	1.44	1.01, 2.06	0.045	1.76	0.91, 3.40	0.096	1.32	0.97, 1.82	0.068
	FinnGen	75	275	0.658	1.35	1.07, 1.71	0.011	1.59	0.98, 2.56	0.064	1.31	1.04, 1.70	NA
Coronary revascularization	FinnGen	75	175	0.694	1.36	0.82, 2.25	0.229	1.47	0.60, 3.62	0.406	1.58	1.04, 2.39	0.036
Ischemic stroke	Consortium	74	183	0.415	0.82	0.61, 1.10	0.191	0.77	0.46, 1.29	0.325	0.77	0.61, 0.96	0.023
	UKBB	75	106	0.565	1.12	0.72, 1.72	0.621	1.32	0.70, 2.48	0.395	0.98	0.71, 1.48	0.915
	FinnGen	75	114	0.198	0.70	0.45, 1.10	0.120	0.73	0.38, 1.38	0.335	0.76	0.54, 1.07	0.118
Large artery stroke	Consortium	74	142	0.964	0.86	0.41, 1.80	0.681	1.60	0.51, 4.98	0.419	8.67	2.53, 273	0.009
Small vessel stroke	Consortium	74	84	0.806	1.31	0.67, 2.59	0.432	1.17	0.52, 2.63	0.699	1.73	0.72, 3.25	0.269
Cardioembolic stroke	Consortium	74	108	0.076	0.55	0.32, 0.95	0.033	0.45	0.21, 0.95	0.041	0.70	0.37, 1.22	0.174
Transient ischemic attack	UKBB	75	88	0.498	0.83	0.52, 1.35	0.461	0.88	0.47, 1.63	0.684	0.95	0.66, 1.35	0.736
	FinnGen	75	106	0.799	0.91	0.52, 1.60	0.746	0.79	0.38, 1.64	0.526	0.62	0.33, 0.98	0.046
Aortic aneurysm	UKBB	75	93	0.466	0.67	0.31, 1.49	0.328	0.93	0.31, 2.82	0.905	0.39	0.06, 0.87	0.027
	FinnGen	75	101	0.363	0.62	0.30, 1.29	0.203	0.60	0.24, 1.49	0.274	0.49	0.27, 1.23	0.126
Peripheral artery disease	UKBB	75	149	0.911	1.18	0.70, 1.99	0.540	1.18	0.47, 2.97	0.720	1.13	0.79, 1.70	0.517
	FinnGen	75	99	0.759	1.05	0.59, 1.86	0.866	1.46	0.70, 3.03	0.314	1.27	0.70, 1.88	0.421
Chronic kidney disease	Consortium	75	207	0.372	1.05	0.80, 1.39	0.705	1.35	0.81, 2.25	0.251	1.23	0.89, 1.51	0.191
	FinnGen	75	81	0.498	1.18	0.60, 2.29	0.634	0.80	0.34, 1.90	0.613	1.03	0.62, 2.69	0.896
Subarachnoid hemorrhage	Consortium	52	83	0.160	1.13	0.53, 2.37	0.753	1.74	0.59, 5.14	0.321	0.93	0.50, 1.92	NA
	FinnGen	75	81	0.458	0.57	0.17, 1.95	0.372	0.47	0.11, 2.02	0.313	0.04	0.01, 0.21	1.32E-04
Venous thromboembolism	UKBB	75	319	0.224	0.73	0.45, 1.18	0.205	0.55	0.17, 1.72	0.306	0.62	0.44, 0.90	0.018
	FinnGen	75	570	0.325	0.76	0.56, 1.03	0.080	0.66	0.26, 1.71	0.399	0.81	0.66, 1.02	0.076
Deep venous thrombosis	UKBB	75	274	0.124	0.38	0.20, 0.74	0.004	0.30	0.07, 1.30	0.112	NA	NA	NA
	FinnGen	75	404	0.295	0.68	0.45, 1.01	0.059	0.56	0.20, 1.51	0.253	0.70	0.52, 0.97	0.036
Pulmonary embolism	UKBB	75	163	0.417	1.15	0.61, 2.17	0.668	0.75	0.23, 2.45	0.638	0.78	0.15, 3.71	0.687
	FinnGen	75	375	0.513	1.01	0.67, 1.53	0.943	0.81	0.28, 2.41	0.712	1.00	0.73, 1.40	0.999

CI, confidence interval; NA, not available; OR, odds ratio.

Supplementary Table 8. Comparison of associations for genetically predicted FG, 2hGlu, and FI in the main analysis and the sensitivity analysis using SNP-glycemic trait estimates without adjustment for body mass index

	**Main analysis**			**Sensitivity analysis**		
	**OR**	**95% CI**	**P**	**OR**	**95% CI**	**P**
**FG**						
Coronary artery disease	1.18	1.04, 1.33	0.009	1.36	1.06, 1.73	0.014
Angina	1.23	1.07, 1.41	0.004	1.36	1.04, 1.8	0.027
Coronary atherosclerosis	1.33	1.17, 1.5	0.000	1.43	1.11, 1.85	0.006
Coronary revascularization	1.33	1.02, 1.75	0.038	1.38	0.81, 2.34	0.234
Ischemic stroke	1.15	1.02, 1.29	0.022	1.08	0.88, 1.33	0.452
Large artery stroke	1.71	1.13, 2.59	0.011	2.70	1.27, 5.74	0.010
Small vessel stroke	1.18	0.86, 1.61	0.301	1.21	0.68, 2.17	0.520
Cardioembolic stroke	1.15	0.87, 1.52	0.319	1.10	0.7, 1.71	0.689
Transient ischemic attack	1.00	0.85, 1.18	0.999	0.93	0.71, 1.24	0.636
Aortic aneurysm	0.84	0.64, 1.11	0.217	0.87	0.54, 1.41	0.573
Peripheral artery disease	0.75	0.54, 1.04	0.089	0.80	0.42, 1.54	0.514
Chronic kidney disease	1.42	1.16, 1.76	0.001	1.82	1.21, 2.73	0.004
Subarachnoid hemorrhage	1.13	0.97, 1.32	0.108	1.48	1.11, 1.96	0.007
Venous thromboembolism	1.00	0.73, 1.36	0.987	0.92	0.75, 1.13	0.439
Deep venous thrombosis	1.04	0.73, 1.47	0.830	0.91	0.68, 1.22	0.521
Pulmonary embolism	0.99	0.71, 1.38	0.966	0.94	0.7, 1.28	0.711
**2hGlu**						
Coronary artery disease	1.19	1.07, 1.34	0.002	1.32	1.18, 1.46	3.38E-07
Angina	1.18	1.05, 1.34	0.007	1.43	1.23, 1.65	1.67E-06
Coronary atherosclerosis	1.14	1.01, 1.29	0.035	1.28	1.16, 1.42	7.30E-07
Coronary revascularization	1.26	0.97, 1.63	0.081	1.69	1.25, 2.29	0.001
Ischemic stroke	1.09	0.99, 1.2	0.070	1.11	0.96, 1.27	0.156
Large artery stroke	1.60	1.11, 2.3	0.012	1.83	0.99, 3.38	0.052
Small vessel stroke	1.45	1.14, 1.86	0.003	1.38	0.97, 1.97	0.073
Cardioembolic stroke	1.21	0.89, 1.63	0.224	1.14	0.85, 1.53	0.397
Transient ischemic attack	1.01	0.9, 1.14	0.868	1.07	0.85, 1.35	0.552
Aortic aneurysm	0.82	0.68, 0.98	0.031	0.78	0.54, 1.11	0.164
Peripheral artery disease	1.24	0.98, 1.58	0.071	1.08	0.78, 1.5	0.647
Chronic kidney disease	1.35	1.12, 1.62	0.002	1.51	1.03, 2.19	0.033
Subarachnoid hemorrhage	1.01	0.9, 1.13	0.871	0.96	0.76, 1.22	0.739
Venous thromboembolism	0.85	0.73, 0.99	0.039	0.80	0.64, 1.02	0.067
Deep venous thrombosis	0.95	0.77, 1.17	0.653	0.76	0.62, 0.95	0.015
Pulmonary embolism	0.74	0.62, 0.89	0.001	0.86	0.69, 1.07	0.173
**FI**						
Coronary artery disease	1.88	1.45, 2.44	1.80E-06	1.75	1.05, 2.92	0.032
Angina	1.84	1.37, 2.49	6.14E-05	1.74	1.04, 2.92	0.036

Coronary atherosclerosis	1.91	1.48, 2.46	5.14E-07	0.55	0.25, 1.22	0.142
Coronary revascularization	2.95	1.58, 5.5	0.001	1.51	0.91, 2.51	0.108
Ischemic stroke	1.32	1.05, 1.65	0.016	1.58	0.86, 2.88	0.138
Large artery stroke	1.41	0.6, 3.33	0.431	2.57	0.95, 6.95	0.063
Small vessel stroke	2.30	1.04, 5.09	0.039	2.31	0.62, 8.65	0.213
Cardioembolic stroke	0.85	0.53, 1.36	0.499	0.64	0.35, 1.15	0.134
Transient ischemic attack	1.29	0.95, 1.75	0.105	1.39	0.82, 2.35	0.217
Aortic aneurysm	1.51	0.86, 2.66	0.154	0.72	0.45, 1.17	0.183
Peripheral artery disease	1.10	0.56, 2.14	0.785	1.25	0.84, 1.87	0.272
Chronic kidney disease	1.91	1.25, 2.91	0.003	1.89	0.51, 7.04	0.345
Subarachnoid hemorrhage	1.48	1.13, 1.95	0.005	1.64	0.56, 4.75	0.366
Venous thromboembolism	0.96	0.63, 1.47	0.853	1.47	0.26, 8.37	0.661
Deep venous thrombosis	1.06	0.64, 1.76	0.828	1.18	0.53, 2.62	0.684
Pulmonary embolism	0.95	0.6, 1.49	0.812	0.95	0.51, 1.74	0.857

CI indicates confidence interval; OR, odds ratio.

Supplementary Table 9. Associations of genetically predicted glycemic traits with atherosclerotic and thrombotic outcomes in multivariable Mendelian randomization analysis with mutual adjustment

Exposure	Outcome	OR	95% CI	Original P	P_FDR	Significant using an FDR of 0.05?
FG	Coronary atherosclerosis	1.24	1.06, 1.46	7.76E-03	1.24E-01	Suggestive
FG	Chronic kidney disease	1.21	1.01, 1.44	3.36E-02	2.69E-01	Suggestive
FG	Peripheral artery disease	1.27	0.99, 1.61	5.54E-02	2.95E-01	No
FG	Coronary artery disease	1.12	0.97, 1.28	1.30E-01	3.20E-01	No
FG	Ischemic stroke	1.13	0.98, 1.3	1.06E-01	3.20E-01	No
FG	Cardioembolic stroke	1.30	0.94, 1.82	1.15E-01	3.20E-01	No
FG	Subarachnoid hemorrhage	0.76	0.52, 1.1	1.40E-01	3.20E-01	No
FG	Angina	1.10	0.93, 1.29	2.81E-01	5.61E-01	No
FG	Coronary revascularization	1.19	0.83, 1.7	3.36E-01	5.77E-01	No
FG	Venous thromboembolism	1.11	0.88, 1.41	3.61E-01	5.77E-01	No
FG	Small vessel stroke	0.86	0.58, 1.26	4.38E-01	6.37E-01	No
FG	Deep venous thrombosis	1.12	0.8, 1.58	5.01E-01	6.68E-01	No
FG	Large artery stroke	1.16	0.72, 1.87	5.49E-01	6.75E-01	No
FG	Aortic aneurysm	0.94	0.68, 1.32	7.41E-01	8.47E-01	No
FG	Pulmonary embolism	0.97	0.7, 1.35	8.60E-01	9.17E-01	No
FG	Transient ischemic attack	0.99	0.82, 1.21	9.46E-01	9.46E-01	No
2hGlu	Peripheral artery disease	1.34	1.18, 1.53	1.18E-05	1.89E-04	Yes
2hGlu	Coronary artery disease	1.17	1.08, 1.25	3.39E-05	2.71E-04	Yes
2hGlu	Small vessel stroke	1.44	1.17, 1.77	6.93E-04	3.69E-03	Yes
2hGlu	Ischemic stroke	1.13	1.05, 1.22	1.87E-03	7.50E-03	Yes
2hGlu	Large artery stroke	1.44	1.1, 1.87	7.33E-03	2.35E-02	Yes
2hGlu	Angina	1.12	1.03, 1.23	1.05E-02	2.81E-02	Yes
2hGlu	Subarachnoid hemorrhage	0.80	0.64, 0.98	3.49E-02	7.98E-02	Suggestive
2hGlu	Coronary atherosclerosis	1.09	1, 1.19	5.86E-02	1.17E-01	No
2hGlu	Aortic aneurysm	0.86	0.72, 1.03	1.01E-01	1.79E-01	No
2hGlu	Coronary revascularization	1.16	0.96, 1.41	1.27E-01	2.04E-01	No
2hGlu	Transient ischemic attack	1.05	0.94, 1.16	4.05E-01	5.89E-01	No
2hGlu	Pulmonary embolism	0.94	0.79, 1.12	5.05E-01	6.73E-01	No
2hGlu	Chronic kidney disease	0.97	0.89, 1.07	5.54E-01	6.81E-01	No
2hGlu	Deep venous thrombosis	1.05	0.87, 1.26	6.11E-01	6.99E-01	No
2hGlu	Venous thromboembolism	1.03	0.9, 1.16	6.89E-01	7.35E-01	No
2hGlu	Cardioembolic stroke	1.03	0.86, 1.24	7.55E-01	7.55E-01	No
FI	Coronary atherosclerosis	1.67	1.3, 2.15	6.80E-05	1.09E-03	Yes
FI	Angina	1.60	1.23, 2.08	4.03E-04	3.22E-03	Yes
FI	Chronic kidney disease	1.52	1.15, 2.01	3.09E-03	1.65E-02	Yes
FI	Coronary artery disease	1.37	1.1, 1.72	5.17E-03	2.07E-02	Yes
FI	Coronary revascularization	2.14	1.22, 3.77	8.07E-03	2.58E-02	Yes
FI	Transient ischemic attack	1.49	1.1, 2.03	1.12E-02	2.98E-02	Yes
FI	Small vessel stroke	2.09	1.13, 3.86	1.92E-02	4.40E-02	Suggestive
FI	Aortic aneurysm	1.77	1.04, 3	3.55E-02	7.10E-02	Suggestive
FI	Ischemic stroke	1.26	1, 1.58	4.89E-02	8.69E-02	Suggestive

FI	Subarachnoid hemorrhage	1.66	0.89, 3.09	1.10E-01	1.76E-01	No
FI	Peripheral artery disease	1.28	0.87, 1.88	2.03E-01	2.95E-01	No
FI	Venous thromboembolism	1.19	0.82, 1.72	3.51E-01	4.68E-01	No
FI	Cardioembolic stroke	0.87	0.51, 1.47	5.99E-01	7.37E-01	No
FI	Large artery stroke	0.95	0.44, 2.05	8.99E-01	9.50E-01	No
FI	Deep venous thrombosis	1.04	0.61, 1.78	8.72E-01	9.50E-01	No
FI	Pulmonary embolism	0.98	0.59, 1.65	9.50E-01	9.50E-01	No
HbA1c	Ischemic stroke	0.80	0.66, 0.98	3.10E-02	4.96E-01	Suggestive
HbA1c	Angina	1.20	0.96, 1.51	1.14E-01	6.08E-01	No
HbA1c	Cardioembolic stroke	0.68	0.43, 1.07	9.19E-02	6.08E-01	No
HbA1c	Coronary artery disease	1.09	0.89, 1.34	3.82E-01	6.62E-01	No
HbA1c	Coronary atherosclerosis	1.13	0.9, 1.41	2.85E-01	6.62E-01	No
HbA1c	Large artery stroke	1.26	0.65, 2.43	4.97E-01	6.62E-01	No
HbA1c	Small vessel stroke	1.21	0.71, 2.05	4.82E-01	6.62E-01	No
HbA1c	Transient ischemic attack	0.91	0.69, 1.19	4.86E-01	6.62E-01	No
HbA1c	Subarachnoid hemorrhage	1.21	0.71, 2.07	4.83E-01	6.62E-01	No
HbA1c	Venous thromboembolism	0.86	0.63, 1.18	3.50E-01	6.62E-01	No
HbA1c	Deep venous thrombosis	0.77	0.48, 1.22	2.64E-01	6.62E-01	No
HbA1c	Pulmonary embolism	1.23	0.78, 1.94	3.64E-01	6.62E-01	No
HbA1c	Aortic aneurysm	0.93	0.58, 1.47	7.42E-01	8.97E-01	No
HbA1c	Chronic kidney disease	0.97	0.76, 1.23	7.85E-01	8.97E-01	No
HbA1c	Peripheral artery disease	0.97	0.69, 1.35	8.42E-01	8.98E-01	No
HbA1c	Coronary revascularization	1.01	0.6, 1.69	9.64E-01	9.64E-01	No

CI, confidence interval; OR, odds ratio.

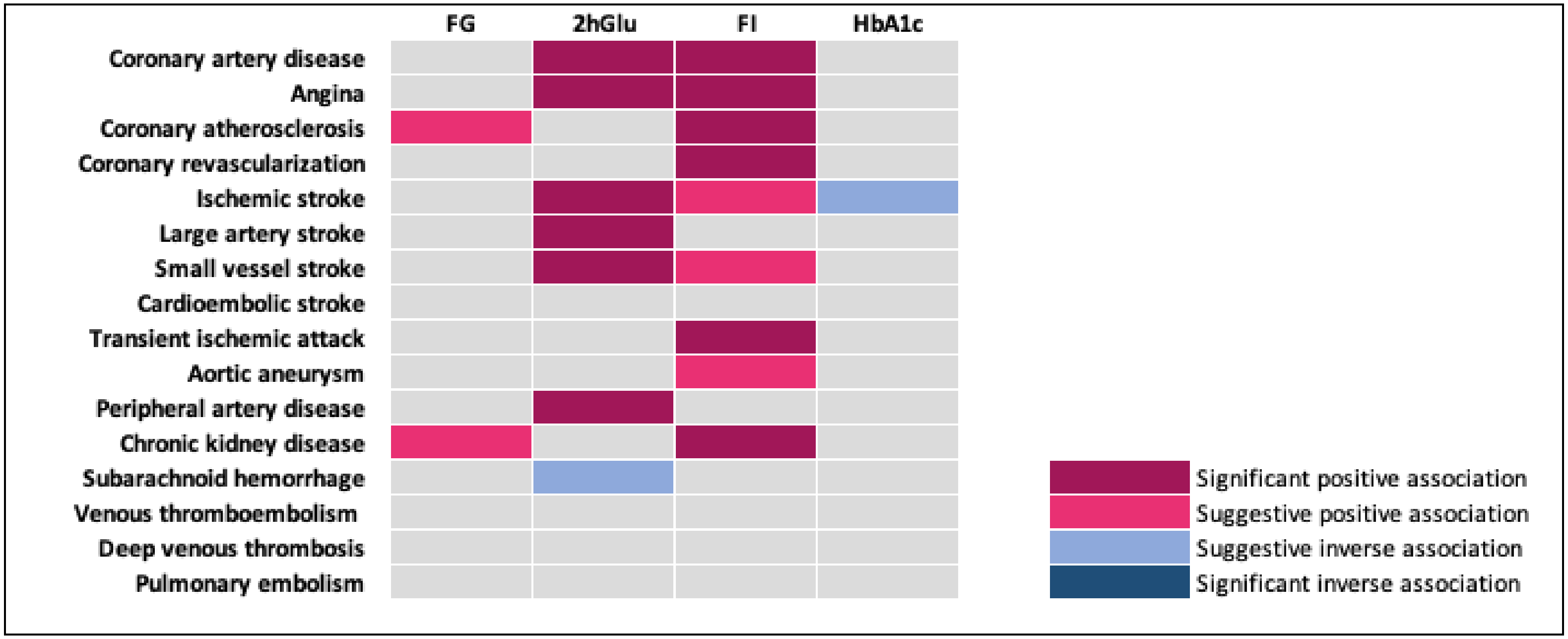

Supplementary Figure 1. Summary of associations of genetically predicted glycemic traits with 12 atherosclerotic and 4 thrombotic outcomes in multivariable Mendelian randomization analysis. 2hGlu, 2-h glucose after an oral glucose challenge; FG, fasting glucose; FI, fasting insulin; HbA1c, glycated hemoglobin.
